# Electronic spectra of ytterbium fluoride from relativistic electronic structure calculations†

**DOI:** 10.1039/d1cp03701c

**Published:** 2021-10-01

**Authors:** Johann V. Pototschnig, Kenneth G. Dyall, Lucas Visscher, André Severo Pereira Gomes

**Affiliations:** Institut für Experimentalphysik, Technische Universität Graz Petersgasse 16 8010 Graz Austria johann.pototschnig@tugraz.at; Department of Chemistry and Pharmaceutical Sciences, Faculty of Sciences, Vrije Universiteit Amsterdam De Boelelaan 1083 NL-1081 HV Amsterdam The Netherlands visscher@chem.vu.nl; Dirac Solutions 10527 NW Lost Park Drive Portland OR 97229 USA diracsolutions@gmail.com; Université de Lille, CNRS, UMR 8523 - PhLAM - Physique des Lasers Atomes et Molécules F-59000 Lille France andre.gomes@univ-lille.fr

## Abstract

We report an investigation of the low-lying excited states of the YbF molecule-a candidate molecule for experimental measurements of the electron electric dipole moment-with 2-component based multi-reference configuration interaction (MRCI), equation of motion coupled cluster (EOM-CCSD) and the extrapolated intermediate Hamiltonian Fock-space coupled cluster (XIHFS-CCSD). Specifically, we address the question of the nature of these low-lying states in terms of configurations containing filled or partially-filled Yb 4f shells. We show that while it does not appear possible to carry out calculations with both kinds of configurations contained in the same active space, reliable information can be extracted from different sectors of Fock space-that is, by performing electron attachment and detachment IHFS-CCSD and EOM-CCSD calculation on the closed-shell YbF^+^ and YbF^−^ species, respectively. From these calculations we predict *Ω* = 1/2, 3/2 states, arising from the 4f^13^σ^2^_6s_, 4f^14^5d^1^/6p^1^, and 4f^13^5d^1^σ^1^_6s_ configurations to be able to interact as they appear in the same energy range around the ground-state equilibrium geometry. As these states are generated from different sectors of Fock space, they are almost orthogonal and provide complementary descriptions of parts of the excited state manifold. To obtain a comprehensive picture, we introduce a simple adiabatization model to extract energies of interacting *Ω* = 1/2, 3/2 states that can be compared to experimental observations.

## Abstract 2

Potential energy curves for the YbF obtained by relativistic electronic structure methods are presented. Due to the difficulties of describing this system separate computations for open and closed f-shells were necessary.

## Graphical abstract

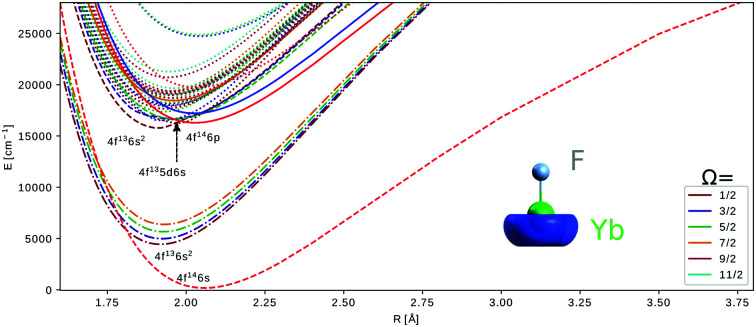

## 1 Introduction

In a previous paper,^1^ we introduced all-electron relativistic basis sets for the lanthanides (La–Lu) and discussed their performance for the determination of spectroscopic constants for the ground state of ytterbium fluoride (YbF), an open-shell molecule with a ^2^Σ^+^ ground state. This molecule has received a fair amount of experimental and theoretical attention because of its potential application in the observation of parity-violating interactions^2–4^*via* determination of the electric dipole moment of the electron (eEDM)-see, for instance ref. 4–11 and references therein). There is also some interest in the Yb atom, cation and dimer in connection to ultracold physics.^12–14^ An example^15^ is the parity violation observed in the Yb atom.

A noteworthy finding in our previous work was the sensitivity of coupled cluster calculations to the basis set in use and, indirectly, to the amount of electron correlation recovered in the calculations. We observed a spike in the values of the *T*_1_ diagnostic around the ground state equilibrium geometry, so it appears that the perturbative treatment of triple excitations in the CCSD(T) calculations breaks down in this region of the potential energy curves unless there is enough flexibility in the correlation treatment. The same was recently observed by Pasteka *et al.*^16^ for the nuclear quadruple coupling constant. This suggests the existence of a low lying perturbing state, which we want to investigate further in the current work.

Experimental^17,18^ and previous theoretical^19–21^ investigations suggest that in the ground state the unpaired electron is located in a σ_s_ orbital with dominant contributions from the 6s orbital of Yb, corresponding to a Yb(4f^14^σ^1^_6s_)F configuration. This ^2^Σ_1/2_ state ground state was studied in greater detail by combining microwave and optical spectroscopy for the odd ^171^Yb isotope.^22^

Experimentally,^18^ the lowest excited state observed is assigned as ^2^Π_1/2_, with an energy of 18 106 cm^−1^, while the ^2^Π_3/2_ component is found at 19 471 cm^−1^, yielding a spin–orbit splitting of 1365 cm^−1^ of this spin–orbit split A ^2^Π state. The lower component will be denoted 3_1/2_ in the current work. Experiments indicate a perturbation of its vibrational levels,^18,23–25^ which was attributed to the presence of a perturbing state (denoted by 4_1/2_ here) found at 18 705 cm^−1^.^18^ This perturbing 4_1/2_ state is sometimes referred to as [18.6]_1/2_ by experimentalists^18,23^ (energy in cm^−1^ divided by 10^3^ in the square brackets, and *Ω*-value as subscript). The mixing of these two *Ω* = 1/2 states gives rise to states designated as [557] and [561] (the values in square brackets referring to transition energies in Thz from the vibronic ground state) with transition energies of 18 574 and 18 699 cm^−1^, respectively.^24,25^ These two states are of importance for laser cooling schemes that have been investigated^26^ and tested^25^ with the purpose of realizing high-accuracy measurements of YbF at very low temperatures. Besides these first excited states, Smallman^26^ investigated also two not yet fully characterized mixed states, [574](≈19 150 cm^−1^) and [578](≈19 280 cm^−1^) at higher energies. These can be compared with the *Ω* = 3/2 state at 19 471 cm^−1^ found earlier by Dunfield,^18^ which will be denoted 2_3/2_ in the current work. Uttam *et al.*^27,28^ furthermore measured additional unidentified higher bands at about 23 035, 23 256 and 26 015 cm^−1^, which they denoted as *C*_1_, *C*_2_ and *D*, respectively.

Theoretically, excited states arising from the Yb(4f^14^6p^1^)F and Yb(4f^14^5d^1^)F configurations were considered by Nayak and Chaudhuri^6^ with RAS-CI based on 4-component spinors, yielding the A^2^Π_1/2_ (3_1/2_), A^2^Π_3/2_ (2_3/2_), and a ^2^Σ_1/2_ state. Earlier multireference CI calculations by Dolg *et al.*^19^ furthermore indicate the possibility of low-lying *Ω* = 1/2 states arising from the 
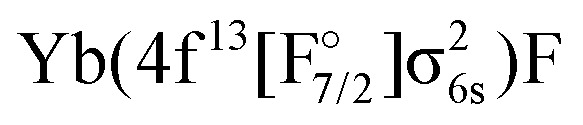
 or 
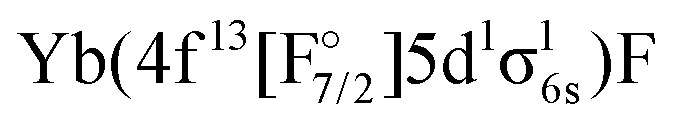
 configurations, to be lying below or close to the Yb(4f^14^6p^1^)F states. This was also found in the DFT calculations of Liu *et al.*^20^ who place excited states arising from the 
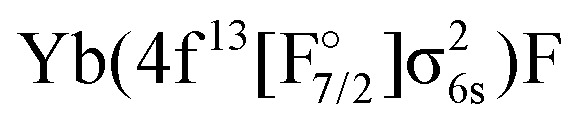
 configuration in the range from 9000 to 15 000 cm^−1^ relative to the Yb(4f^14^σ^1^_6s_)F ground state. These findings make it of interest to explicitly consider the configuration interaction between the f^13^ and f^14^ configurations in the Yb atom.^29^

The vibronic states are additionally split due to hyperfine interactions. In atomic experiments they were measured for the ground and excited states^9,22,30–33^ also using Zeeman spectroscopy.^34^ The hyperfine interaction of the atom^35–37^ and molecule^38^ were studied theoretically, and should have similar uncertainties to the contribution of the eEDM to the spectrum due to the similarity of the matrix elements. Recently, uncertainties of the hyperfine constants arising in relativistic coupled cluster computations have been studied.^39^

It is clear from the above that a proper description of the Yb atom and the YbF molecule requires an accurate treatment of both spin–orbit coupling and electron correlation, for ground as well as excited states. A popular approach is the so-called two-step approach to spin–orbit coupling (SOC), in which electron correlation methods based on non-relativistic or scalar relativistic Hamiltonians are used to obtain excited state energies, that are in turn used to dress a spin–orbit configuration interaction (SOCI) matrix. This approach can yield quite accurate spin–orbit coupled states, but results are particularly sensitive to the number of spin-free states serving as a basis for the SOCI step.^40–46^ An alternative is to include SOC already at the mean-field level, and use fully SO-coupled molecular spinors to construct the correlated wave functions.^47^ This can be done with four-component Hamiltonians, as done for the ground^1^ and excited states^6,37,48,49^ of YbF, or with more computationally efficient two-component Hamiltonians based on the eXact 2-Component (X2C) approach,^50–57^ in which a transformation to decouple the positive and negative energy states of the Dirac Hamiltonian can be carried out in matrix form, yielding the same positive energy spectrum as the original 4-component Hamiltonian. More details can be found in the recent review by Liu.^47^ Among the different X2C flavors, we can distinguish two main strategies for the decoupling, which is performed based on: (i) the one-electron Dirac Hamiltonian prior to the mean-field step,^54,57^ and for which two-electron spin–orbit contributions due to the untransformed two-electron potential are included *via* atomic mean-field contributions calculated with the AMFI code^41,58,59^ (X2C-AMFI); (ii) after a converged 4-component mean-field calculation on atoms^52,53,56^ or molecules^55^ (^2^DC^M^). Recent benchmarks show that ^2^DC^M^ calculations closely reproduce equivalent 4-component ones for valence^60^ or core^61^ states.

Moreover, the aforementioned calculations for the excited states of YbF have mostly employed multireference CI (MRCI) approaches. While these can provide great flexibility in capturing static correlation, it remains the case that dynamical correlation is better accounted for with coupled cluster approaches. Among the coupled cluster singles and doubles (CCSD) approaches for excited states, we have the equation of motion (EOM-CCSD) method as well as Fock-space (IHFS-CCSD) methods,^62^ of which the single electron attachment, detachment, and singly excited states variants are the most commonly used. The two approaches have been found to yield very accurate results in general and in particular for calculations with relativistic Hamiltonians as discussed elsewhere (see ref. 60 and references therein).

The first goal of this work is therefore to go beyond the investigations performed to date in the literature, and apply the relativistic EOM-CCSD and IHFS-CCSD approaches to describe the low-lying excited states of YbF. For such states, where the most important excited state configurations appear have a single open-shell character (4f^14^σ^1^_6s_, 
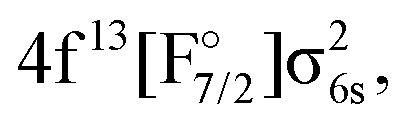
 4f^14^5d^1^, 
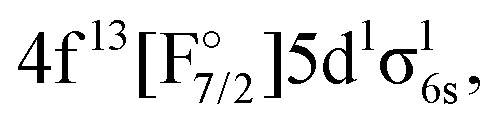
 4f^14^6p^1^), these coupled cluster approaches are in principle applicable, provided one starts from closed shell configurations such as Yb(4f^14^σ^2^_6s_)F^−^ or Yb(4f^14^)F^+^. Additionally, we assess the performance of relativistic MRCI with respect to the coupled cluster methods. Our second goal is to confirm whether any low-lying state is close enough to the ground state to perturb the latter, and explain the anomalous behavior observed in the open-shell ground-state calculations in the literature.

## 2 Computational details

All relativistic electronic structure calculations were performed with a development version of the Dirac program suite^63^ (revision 6e10c5d3), employing for Yb the valence double-zeta (24s19p13d9f2g), triple-zeta (30s24p18d14f4g2h) and quadruple-zeta (35s30p19d16f6g4h2i) basis sets from the previous work,^1^ along with the matching augmented correlation-consistent (aug-cc-pVnZ, *n* = 2, 3, 4) basis sets of Dunning^64^ for F. All basis sets were kept uncontracted, with the small component basis generated by restricted kinetic balance. In addition to these individual basis sets, we have used the calculations with triple- and quadruple-zeta sets to construct extrapolations to the complete basis set limit (*E*_∞_) for the underlying potential energy curves, using the relation^65^1
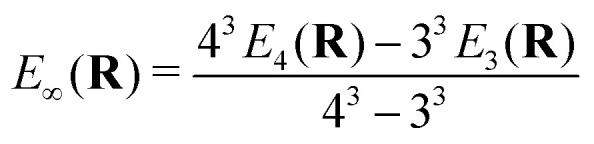
where the subscripts denote the cardinal numbers for the basis sets and *E*_*n*_(**R**) the energy for a given geometry and electronic structure method for a basis of cardinal number *n*(= 2, 3, 4).

In the coupled cluster computations the ^2^DC^M^ Hamiltonian^54,55^ was applied, all two-electron integrals over small component (S) basis sets (*i.e.* the so-called (SS|SS)-type integrals) appearing in the SCF step have been replaced by a simple correction.^66^ In order to account for spin–orbit coupling and other relativistic effects the X2C-AMFI Hamiltonian was employed for the Kramers-restricted configuration interaction (KRCI) method.

Spectroscopic constants (*r*_e_, *D*_e_, *ω*_e_ and *ω*_e_*χ*_e_) were determined from a Morse potential fit in the vicinity of the potential energy minima. The potential energy curves were determined for bond lengths between 1.6 Å and 2.3 Å spaced by 0.02 Å and additional points with larger spacing up to 3.5 Å. In the calculation of *D*_e_ the asymptotic dissociation limit is calculated from the energies of the isolated neutral atoms, F in the ^2^P_3/2_ state and Yb in the ^1^S_0_ state.

The dataset associated with this manuscript (outputs from calculations, codes to extract and process information from these, and code to obtain the spectroscopic constants) is provided in ref. 67.

### 2.1 Kramers-restricted configuration interaction

For YbF we first consider Kramers-restricted configuration interaction (KRCI) based on an average-of-configuration Hartree–Fock approach (AOC-SCF).^68^ This method was employed in order to treat the open shells, where one or two valence electrons were distributed over the s- and d-orbitals and the f-shell was either completely filled or contained one hole, depending on the states of interest. The AOC-SCF reference wave function in the KRCI computation is occupied according to a definition given by a generalized active space (GAS).^69^ In this approach the Hamiltonian is computed for all allowed configurations and then diagonalized. The GAS space was defined by a f-shell which was completely filled or contained one hole and one or two electrons distributed over 29 orbitals.

### 2.2 Equation-of-motion coupled cluster

The first approach we use to describe the dynamical correlation that is largely missing in KRCI is EOM-CCSD, which can give access to electronic states of different kinds, depending on the single determinant wave function that is chosen as the starting point. In it, the CCSD amplitudes are determined for the chosen ground state in the first step, and subsequently the similarity transformed Hamiltonian is constructed using these amplitudes and the desired states are generated by an operator that either removes or adds an electron.

The first set of states was obtained by electron attachment on Yb(4f^14^F^+^ ion, where the HOMO (σ_6s,1/2_) of YbF was initially empty. This computation on the (0h,1p) sector of Fock space yielded states with 4f^14^ and a valence electron in the σ_6s_, d or p orbital. This means that, in the process of obtaining the potentials for the ground and excited states of YbF, we immediately obtain energies of CCSD quality for YbF^+^, and therefore vertical ionization potentials (IP) at each geometry.

Another set of states was obtained by ionizing the Yb(4f^14^σ^2^_6s_)F^−^ anion, where the HOMO (σ_6s,1/2_) of YbF was initially doubly occupied. States arising from the Yb(4f^14^σ^1^_6s_)F, 
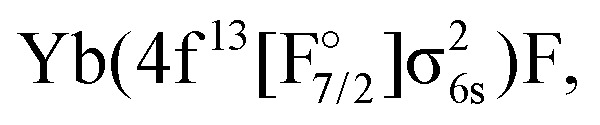

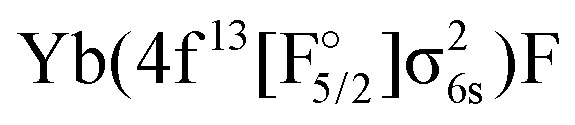
 and Yb(4f^14^σ^2^_6s_)F(2p^5^) configurations were obtained by considering the (1h,0p) sector of Fock space. This means that, in the process of obtaining the potentials for the ground and excited states of YbF, we immediately obtain energies of CCSD quality for YbF^−^, and therefore vertical electron affinities (EA) at each geometry. We note that states arising from the (2h,1p) and (1h,2p) manifolds are also accessible from EOM-IP and EOM-EA calculations, though the energy of electronic states determined by such configurations will be less accurate than states dominated by single detachment or attachment configurations.

The EOM-CCSD electronic states are obtained by an iterative diagonalization (Davidson) procedure in which only the energies of a certain number of the lowest states are determined. For the IP-EOM-CCSD we obtained 16 *Ω* = 1/2, 8 *Ω* = 3/2, 6 *Ω* = 5/2 and 2 *Ω* = 7/2 states, whereas for EA-EOM-CCSD we obtained 8 *Ω* = 1/2, 6 *Ω* = 3/2, 4 *Ω* = 5/2 and 2 *Ω* = 7/2 states.

As transition moments are not yet available for the EOM-CCSD implementation in Dirac, we have only obtained the potential energy curves. These are nevertheless useful since, by not requiring the definition of model spaces or the use of an extrapolation procedure, they serve as a cross validation of the IHFS-CCSD calculations below.

### 2.3 Fock-space coupled cluster

Fock-space coupled cluster^70^ (FS-CCSD) is our second approach to include dynamical correlation in the electronically excited states. Here it was employed in a similar fashion to EOM-CCSD, starting from YbF^+^ or YbF^−^ and proceeding to the (0h,1p) and (1h,0p) sectors of Fock space, respectively. For FS-CCSD a model space is defined by selecting a number of occupied and virtual orbitals and how many electrons are added and removed. The matrix for this subspace is constructed and subsequently diagonalized, thus yielding all states within the chosen model space, in this case states arising from single electron attachment (EA) or single electron detachment (IP). This method requires solving first for the underlying sectors, starting with (0h,0p), which corresponds to CCSD. Due to computational constraints, we have truncated the virtual space so that 117, 230 and 296 orbitals were used in the double-, triple- and quadruple-zeta CCSD calculations, respectively.

The separation into a model and external space leads to the appearance of the so-called intruder states, a well-known difficulty with Fock-space coupled cluster and other effective Hamiltonian approaches, that can be dealt with in many cases by the intermediate Hamiltonian (IH) Fock-space coupled cluster (IHFS-CCSD) method.^71,72^

The IH approach was employed to compute Yb(4f^14^{σ_6s_,6p,5d…}^1^)F states starting from YbF^+^. The active P space in such calculations contained about 50 spinors varying slightly with bond distance and basis set. Of these 26 spinors are always present in the model (*P*_m_) space, whereas the remaining active spinors are placed in the intermediate (*P*_I_) space. Due to using the (0h,1p) sector for the cation, states arising from configurations where the Yb 4f shell is partially filled (such as 
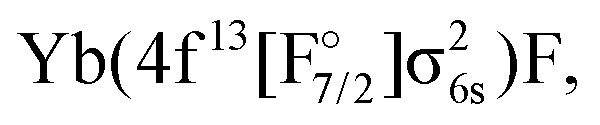

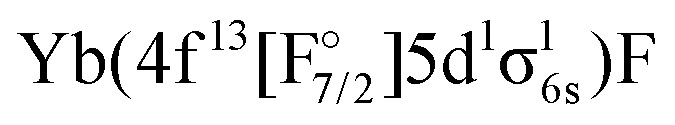
 or 
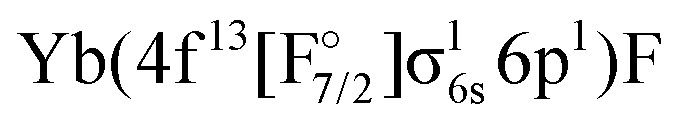
) are not accessible in this calculation.

The approach outlined above was, however, not enough to avoid divergence in the computation of the (1h,0p) sector using the anion as a reference. Therefore, the extrapolated intermediate Hamiltonian (XIH) Fock-space coupled-cluster approach^73^ (XIHFS-CCSD) was applied using the same shifts as in ref. 73. Values of 0.1 and 0.2 Hartree were selected if one of the holes is not in the model space. These shifts were doubled for two holes outside the model space. Using the determined energies an extrapolation to the system without shifts was performed. The model (*P*_m_) space in these computations contained 22 spinors, the intermediate (*P*_I_) space about 24 spinors depending on the bond distance and basis set. Since we start out from the anion and only allow holes, only Yb(4f^14^σ^1^_6s_)F, 
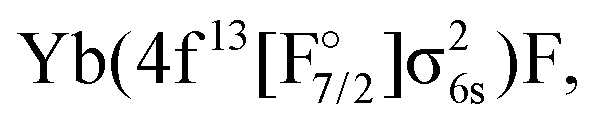

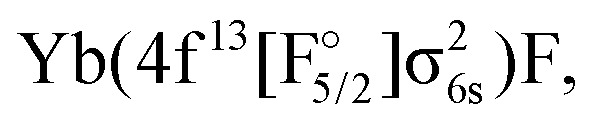
 and Yb(4f^14^σ^2^_6s_)F(2p^5^) configurations are accessible in this computation.

Combining the two sectors allows us to get different excited states of YbF, although there are limitations. Firstly, the interactions between configurations with open f-shell and the ones with an electron in the p- or d-shell are not included, since they will be obtained for different sectors of Fock space. This interaction will nevertheless be treated with a simple adiabatization approach, described in Sections 2.4 and 3.6. Secondly, configurations such as 
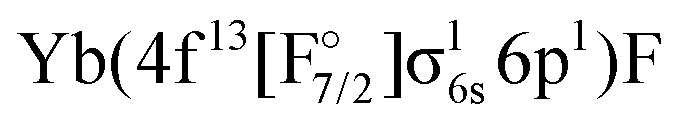
 or 
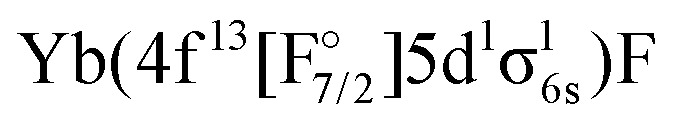
 are not included in the current treatment. This limitations is not as significant because these states have higher energies than the ones we are interested in. Both of these problems could be dealt with by using the (1h,1p) sector, but this goes beyond the current work as convergence is very unstable for this sector and it requires the use of an open-shell reference.

### 2.4 Adiabatization of electronic states

As we separated the computations of states with 4f^13^ and 4f^14^ character, these states cannot interact with each other, and states with the same *Ω* value cross although they should have an avoided crossing. In order to correct this deficiency we considered a simple adiabatization model, in which we set up and diagonalize the following matrix for each *Ω* value:2
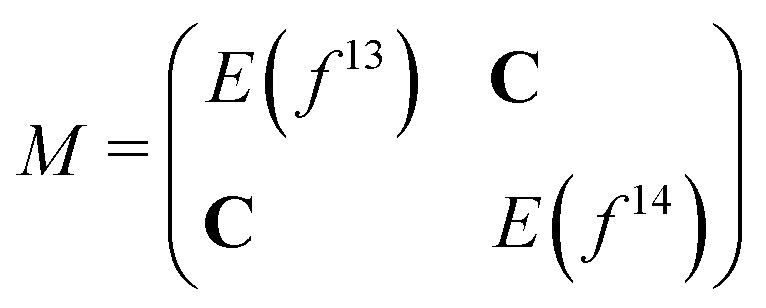
where **C** is a matrix where every entry is a coupling constant (whose value is kept constant for all states and geometries considered; we have investigated values of 0.01, 0.001 and 0.0001 a.u.), *E* are matrices with the eigenvalues of the different electronic states on the diagonal. The potential curves were computed for different coupling constants and the results are shown in Section 3.6. We note that since the ground state energy, associated with the Yb(4f^14^σ^1^_6s_)F configuration, appears in both coupled cluster approaches, we have only considered one such energy. As we shall see in the discussion, this is valid in the region between 1.8 Å and 2.5 Å, since for these distances the ground-state energies from IP and EA calculations are nearly identical.

## 3 Results and discussion

We start our discussion with the electronic transitions of the atomic Yb^+^ cation, before moving on to the YbF molecule. This is because the cation's electronic structure is similar to the Yb in YbF since, due to the large electronegativity of fluorine, one electron is almost completely removed from the Yb atom.

Subsequently, the potential energy curves for Kramers-restricted configuration interaction are presented. A discussion of the coupled cluster approaches follows, with a focus on the comparison of the coupled cluster results for the Fock space and equation-of-motion approach. This section is followed by a presentation of the spectroscopic data for the ground and excited states. In the last part we take a closer look at the mixing of states at around 18 000 cm^−1^ and apply the adiabatization procedure.

### 3.1 Ytterbium cation

As discussed in the introduction, states from both the 4f^13^ and the 4f^14^ configurations are of importance. This is difficult to realize in a balanced manner when using one set of orbitals to describe all states. Any change in the occupation of the 4f-shell will alter the screening of the 5s and 5p orbitals of Yb, resulting in differences between orbital sets optimized for a 4f^13^ or a 4f^14^ configuration. Additionally, the 4f orbitals are very compact and since they are the first f-shell there are no orthogonality conditions limiting the radial expansion or contraction of the orbital. Depictions of the orbitals for both configurations can be found in Table S1 in the ESI.†

These observations help to understand why it turned out to be very difficult to treat both sets of states in the same calculation, which we attempted to do from AOC-SCF on the Yb^+^. We started out by performing AOC-SCF computations on the atom, based on the 4f closed shell configuration. While we obtained the correct ground state configuration, the 
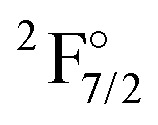
 has an energy of about 46 000 cm^−1^ (over two times higher than the experimental value), and the wrong order for the hole states is observed. If the wave function is optimized for a 4f^13^ configuration, one obtains the 
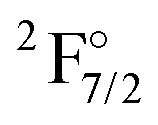
 as the lowest state and finds the true ground state more than 20 000 cm^−1^ higher. Because of these difficulties, the KRCI calculations discussed below all follow the strategy of different orbital sets that is also employed in the subsequent coupled cluster calculations.

Table 1 contains KRCI values of electronic transitions for the cation. The transition energies show deviations of about 10% and the spin–orbit splitting is underestimated for states with a 4f^14^ configuration. The squared transition dipole moment (TDM^2^) of the 
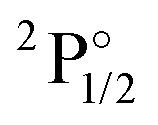
 state is underestimated by about 13%, the one for the 
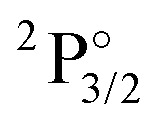
 state overestimated by about 47%. The second set of states with a hole in the f-shell and different distributions of the 2 valence electrons are given in the lower part of Table 1, the energies are relative to the 
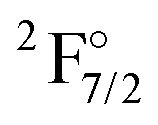
 state. In this case the two valence electrons are distributed over the s- and d-shell. The lowest state with a 
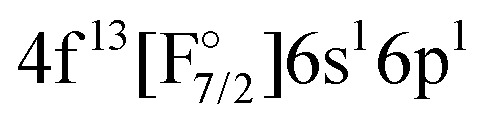
 configuration has a transition energy of 47912.31 cm^−1^ and was not included in the current treatment.

Kramers-restricted configuration interaction transition energies (in cm^−1^), squared transition dipole moments (TDM^2^), and line strength (*S*) for the Yb^+^ cation, the latter two are in atomic units(*e*^2^*a*_0_^2^). Reference values and notation have been taken from the NIST database.^74^ For the 4f^13^ configurations, energies relative to the 
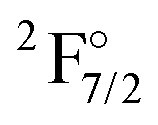
 state are also given. 2z, 3z, 4z, and extr. indicate double, triple, quadruple zeta and extrapolated results, respectively

**Table:** 

State	Conf.	NIST^74^		2z		3z		4z		extr.
		E	S	E	TDM^2^	E	TDM^2^	E	TDM^2^	E
^2^S_1/2_	4f^14^6s^1^	0		0		0		0		0
^2^D_3/2_	4f^14^5d^1^	22 961		23 322	0.0	22 802	0.0	23 606	0.0	24 192
^2^D_5/2_	4f^14^5d^1^	24333		23 882	0.0	23 321	0.0	24 117	0.0	24 698
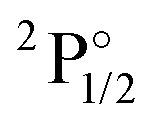	4f^14^6p^1^	27062	6.1	25 210	3.5	24 533	3.8	25 331	3.6	25 914
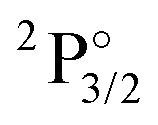	4f^14^6p^1^	30392	11.4	28 104	16.9	27 385	18.9	28 153	17.4	28 712

**Table:** 

State	Conf.	E	Δ*E*	Δ*E*	Δ*E*	Δ*E*	Δ*E*
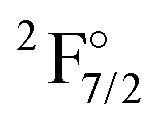	4f^13^6s^2^	21 419	0	0	0	0	0
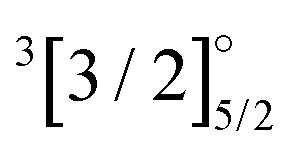	4f^13^5d^1^6s^1^	26 759	5340	4260	5538	4618	3946
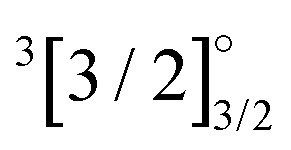	4f^13^5d^1^6s^1^	28 758	7339	6387	7822	7123	6613
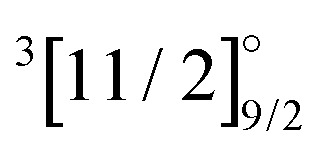	4f^13^5d^1^6s^1^	30 224	8806	8214	9325	8314	7576
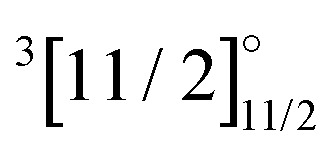	4f^13^5d^1^6s^1^	30 563	9144	8320	9431	8447	7729

The excited states of the Yb atom for the 4f^14^ configuration have already been investigated by relativistic Fock-space coupled cluster^36,37,75,76^ as well as for the cation,^75^ including the transition moment of magnetic transitions.^76^ With our current calculations we can go beyond these studies and investigate the f^13^ configurations as well. Before discussing our IHFS-CCSD calculations for Yb^+^, we focus on the EOM-CCSD excitation energies, shown in Table 2. The EOM-IP-CCSD energies of 4f^13^ states obtained from the extrapolation to the complete basis set limit underestimate the experimental transition energies by around 3000 cm^−1^, whereas the values for 4f^14^ states, obtained with EOM-EA-CCSD are within 1000 cm^−1^ of the experimental values, which yields a quantitative improvement over the KRCI ones for both configurations, even though qualitatively the two methods provide a similar picture. From that and the preceeding discussion, we attribute the relatively lower accuracy for the 4f^13^ to arise from the incomplete account of the relaxation of the wave function upon the creation of the hole in the f shell. Beyond the states presented in Table 2, which are dominated by single electron attachment and detachment, we are able to access states with significant (1h,2p) and (2h,1p) character with EOM-CCSD. These states, available in Table S14 in the ESI,† are about 10 000 cm^−1^ higher in energy than the experimental ones.

**Table:** Transition energies (in cm^−1^) for the Yb^+^ cation, obtained for different basis set with EOM-IP-CCSD (4f^13^) and EOM-EA-CCSD (4f^14^), except for the ground state, for which both methods yield the same configuration and total energy. 2z, 3z, 4z, and extr. indicate double, triple, quadruple zeta and extrapolated results, respectively. Reference values were obtained from the NIST database^74^

State	Conf.	NIST^74^	2z	3z	4z	Extr.
^2^S_1/2_	4f^14^6s^1^	0	0	0	0	0
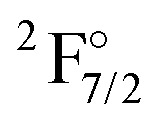	4f^13^6s^2^	21 419	12 054	13 524	16 092	17 966
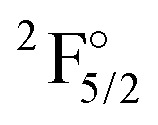	4f^13^6s^2^	31 568	22 629	24 139	26 655	28 491
^2^D_3/2_	4f^14^5d^1^	22 961	24 073	24 209	24 060	23 951
^2^D_5/2_	4f^14^5d^1^	24 333	25 351	25 457	25 341	25 257
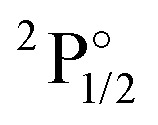	4f^14^6p^1^	27 062	27 539	27 780	27 857	27 913
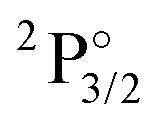	4f^14^6p^1^	30 392	30 954	31 246	31 323	31 380

Finally, our IHFS-CCSD results are presented in Table 3. The transition energies for 4f^14^ configuration reproduce well the experimental ones, with errors below 6%, and only show a small dependence on the basis set. The states arising from the 4f^13^ configuration (
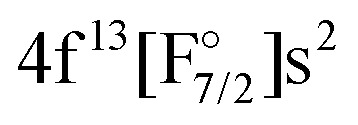
*etc.*), in contrast, show a significant dependence on the basis and a rather slow convergence and underestimate the value by about 30%, which makes them less accurate than the EOM-CCSD ones. This lower accuracy is a consequence of the reduced flexibility in the model spaces, due to the need of adding the 5p-shell just below the 4f-shell to the intermediate space, in order to achieve convergence. These results are in line with the observations of Shee *et al.*,^60^ in that the formal equivalence between EOM-CCSD and IHFS-CCSD for the sectors of Fock space considered depends, in fact, on the flexibility of the main model space.

**Table:** Transition energies for the Yb^+^ cation. Reference values have been obtained from the NIST database,^74^ the computed values were obtained for different basis set sizes with Fock-space coupled cluster

State	Conf.	NIST^74^	2z	3z	4z	Extr.	DCB^75^
^2^S_1/2_	4f^14^6s^1^	0	0	0	0	0	0
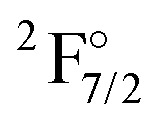	4f^13^6s^2^	21 419	11 087	12 390	13 618	14 514	
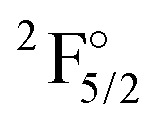	4f^13^6s^2^	31 568	21 631	22 976	24 170	25 042	
^2^D_3/2_	4f^14^5d^1^	22 961	24 058	24 223	24 059	23 938	23 720
^2^D_5/2_	4f^14^5d^1^	24 333	25 336	25 469	25 340	25 246	24 998
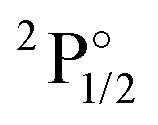	4f^14^6p^1^	27 062	27 518	27 774	27 851	27 907	27 870
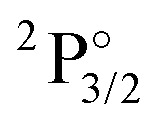	4f^14^6p^1^	30 392	30 934	31 241	31 316	31 371	31 312

Furthermore, the removal of the 5p spinors from the main model space underscores the importance of the 5p for the energetics of the states with a hole in the 4f shell, since by doing so, we undress the contributions from the 5p configurations, and thus prevent them from interacting effectively with 4f^13^ determinants.

### 3.2 Kramers-restricted configuration interaction potential energy curves

**Fig. 1:**
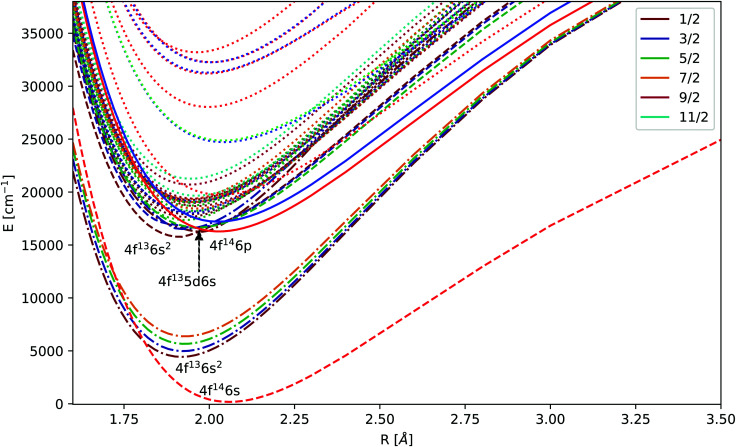
Combination of the sets of KRCI potentials obtained by extrapolating triple and quadruple zeta basis sets. The lowest *Ω* = 1/2 states are denoted by their dominant configuration.

In Fig. 1 the potential energy curves obtained by an approach corresponding to the one used for Yb^+^ are shown for f^14^ and f^13^ configurations. For separate potential energy curves and transition dipole moments we refer the interested reader to the ESI† (Fig. S2–S7).

The potential energy curves have been determined up to 15 Å for the closed and open f-shells. The energy difference in the atom between these states is 21418.75 cm^−1^. Accordingly, the PECs for the hole states were shifted to obtain this separation at this distance. There is still some interaction between ytterbium and fluorine at 15 Å, but the long range behaviour can be expected to be similar for the two configurations (this assumption was checked, see Fig. S8 in the ESI,† for further details). Taking into account the position of the minima, the curvatures, spin–orbit splitting, the avoided crossings and asymptotes the states can be assigned to a dominant configuration, shown in Fig. 1.

Regarding the 4f^14^ manifold, the lowest two excited states in the figure belong to the Yb(4f^14^6p^1^)F configuration, but approach asymptotically the ^2^D_3/2_ state. The asymptote of the next three states is ^2^D_5/2_ corresponding to the Yb(4f^14^5d^1^)F configuration for smaller internuclear separations. For *Ω* = 5/2 the transition dipole moment with the ground state is zero, for the other four the values are shown in Fig. S3 in the ESI.† The first *Ω* = 3/2 and the third *Ω* = 1/2 state have a larger transition dipole moment close to equilibrium, but get close to each other at the largest internuclear separations.

Regarding states of the 4f^13^ manifold, the lowest four states belonging to the 
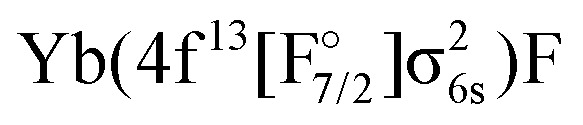
 configuration are well separated from a dense region with a lot of states about 12 000 cm^−1^ higher. Most of these states are of the 
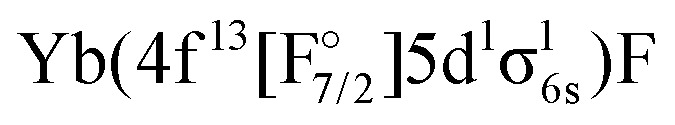
 configuration, with the 
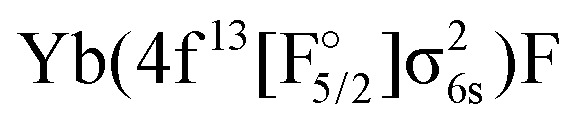
 state slightly higher in energy asymptotically and more strongly bound, resulting in several avoided crossings. For each of the four 
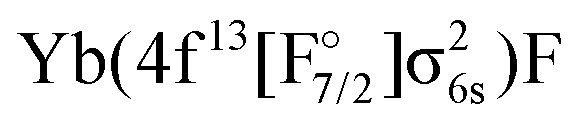
 states the transition dipole moments with higher excited states of varying *Ω* are plotted in Fig. S6 in the ESI.† The transition dipole moments are substantially smaller than the ones for the closed 4f-shells but some of them are non-zero.

An alternative to AOC-SCF for obtaining orbitals for several configurations is multiconfigurational SCF, but similar difficulties as for AOC-SCF in obtaining a balanced description of the 4f^14^ and 4f^13^ states are observed: either the wrong ground state is obtained (if only the hole states are optimized in MCSCF), or the hole states are too high in energy by about 20 000 cm^−1^ (if the ground state is optimized). We also made attempts using state-averaged MCSCF in a non-relativistic quantum chemistry code and observed the same difficulties (see dataset^67^). If the 4f^13^ configurations are excluded one obtains meaningful results, but at the expense of obtaining a 
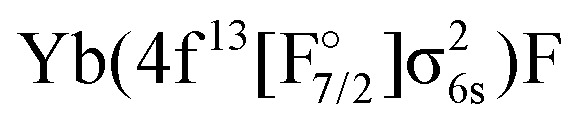
 states too high in energy. If all the states are included, the wrong ground state is obtained.

### 3.3 Coupled cluster potential energy curves

**Fig. 2:**
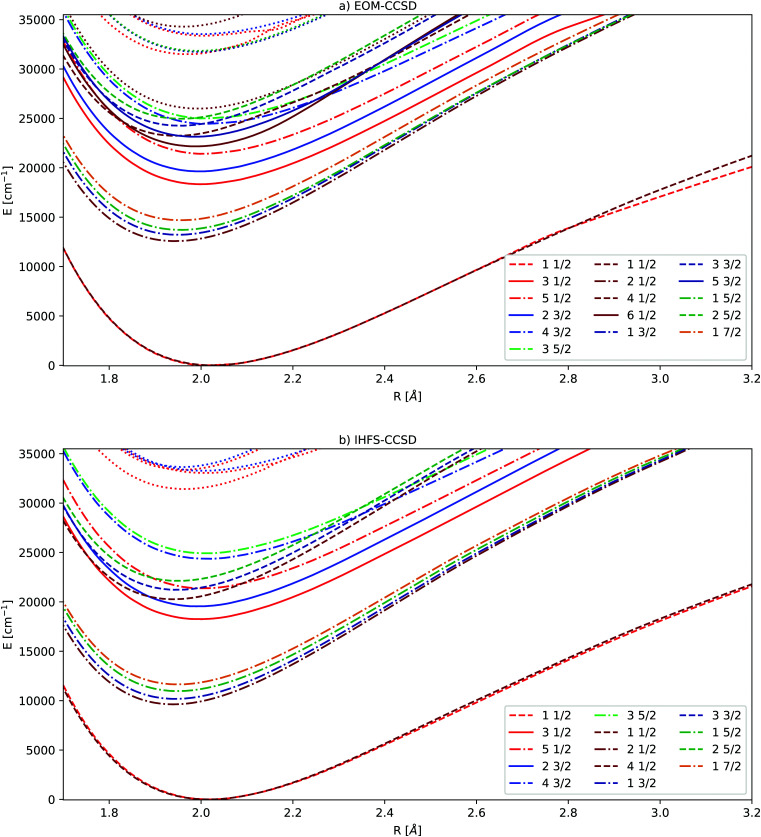
Potential energy curves obtained by extrapolating triple and quadruple zeta basis sets. EOM-CCSD results in the upper part, IHFS-CCSD in the lower part. The *Ω* values of 1/2, 3/2, 5/2 and 7/2 are indicated by the colors red, blue, green and orange. The light colors are used for the (0h,1p) sector, dark ones for (1h,0p). For the states below 30 000 cm^−1^ we employ the same color coding and state notation as in Fig. 1.

The potential energy curves of excited states obtained by the equation-of-motion and Fock space methods are displayed in Fig. 2, the values for the complete basis set limit are shown. The basis set dependence in the molecule is similar to the one observed for Yb^+^: energies for 4f^14^ states depend only weakly on the basis set, while the gap between the ground state and the excited states corresponding to the 
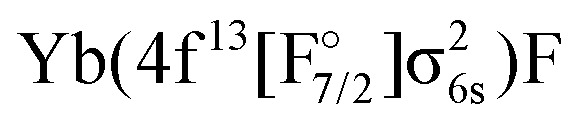
 configuration increases upon improving the basis sets.

While the EOM-CCSD excitations energies of Yb^+^ are closer to the experimental ones, the 
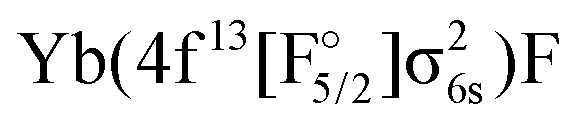
 states are too high to perturb the Yb(4f^14^6p^1^/5d^1^)F PECs. From the extended potential energy curves provided in the ESI† (Fig. S15 and S16), we can observe that the ground state of the non-interacting system (Yb(4f^14^σ^2^_6s_)F(2p^5^)) is repulsive and has a high energy at the equilibrium distance. This results in several avoided crossings being observed at 3, 3.5, and 5 Å.

Since the Yb(4f^14^σ^1^_6s_)F ground state is accessible for both sectors employed in the coupled cluster calculations (Fock space as well as EOM), we can assess the compatibility of the two separate sets of calculations (in the sense of having comparable accuracies) by looking more closely at the differences between the ground states in Fig. 2. From that, we can see that the EOM-CCSD and IHFS-CCSD approaches the curves are on top of each other from the smallest considered internuclear separation up to about 2.8 Å. This assures us that there should not be artifacts in putting together and comparing the calculations on the two sectors.

### 3.4 Dissociation and ionization energies

Since the (1h,0p) and (0h,1p) sectors have been considered in our EOM-CCSD and IHFS-CCSD calculations, we have as a by-product of our calculations the ionization potentials (IP) and electron affinities (EA) for YbF for all computed distances. Therefore these quantities are presented first in Table 4, before proceeding to the spectroscopic constants.

**Table:** Ionization potential (IP), electron affinity (EA) and dissociation energy (*D*_e_) of Yb, F, and YbF. All values in cm^−1^. They are listed for a quadrupole zeta basis set and a basis set extrapolation. The minimum of the potentials were determined using a Morse fit and used to compute the adiabatic values listed here

Quant.	System	KRCI		EOM-CCSD		IHFS-CCSD		Experiment
		4z	Extr.	4z	Extr.	4z	Extr.	
IP	Yb	38 406	39 128	50 735	50 822	50 740	50 837	50 443^74^
IP2	Yb	90 581	90 926	97 919	98 035	97 918	98 040	98 232^74^
IP	F	127 131	126 617	144 153	143 321	144 076	144 703	140 525^74^
EA	F	13 326	11 978	27 279	27 740	27 246	27 759	27 432^77^
IP	YbF	58 478	56 884	48 471	48 578	48 426	49 901	47 700^78^
EA	YbF	7423	7326	9713	9876	9579	8197	
*D*_e_(IP)	YbF	26 059	25 887	43 824	47 782	40 591	40 931	43 260^79^
*D*_e_(EA)	YbF	40 394	39 660	45 534	49 629	40 430	49 053	43 260^79^

Unlike coupled cluster calculations, for KRCI a consistent definition of active spaces is difficult, and its lack of size-consistency results in large deviations from experiment and from the coupled cluster values. For adiabatic electron affinities, for which to the best of our knowledge there are no experimental values, the extrapolated values are 8393 and 8197 cm^−1^ for EOM-CCSD and IHFS-CCSD, respectively. For a distance of 6.5 Å a value of 28 651 cm^−1^ was obtained, which is reasonably close to the electron affinity of fluorine (27 432 cm^−1^).^77^ Corresponding results for the atoms are listed in the table, which allow to calculate the dissociation energies (*D*_e_). They deviate from the experimental values of 43 600 ± 800 cm^−1^ by Kaledin *et al.*^78^ and 43 260 ± 800 cm^−1^ by Yokozeki and Menzinger.^79^ The ionization potentials in Table 4 show acceptable agreement with experimental values.

### 3.5 Spectroscopic constants

The spectroscopic constants for the ground state are now considered. In Table 5 our results are summarized, along those from the literature.

**Table:** Spectroscopic constants for ground state parameters for different approaches. Dissociation energies (*D*_e_), harmonic frequencies (*ω*_e_) and anharmonicity constants (*ω*_e_*χ*_e_) are given in cm^−1^, the equilibrium bond distances (*r*_e_) in Å. For the theoretical results we listed the values obtained by extrapolating triple and quadruple zeta basis sets (CBS)

Method	Ref.	*r*_e_	*ω*_e_	*ω*_e_ *χ*_e_	*D*_e_
KRCI	YbF	2.0829	465	2.40	39 660
EOM-CCSD	YbF^+^	2.0230	511	2.80	49 629
	YbF^−^	2.0250	508	2.53	47 782
IHFS-CCSD	YbF^+^	2.0176	515	2.82	49 053
	YbF^−^	2.0159	513	2.42	40 931

CCSD^1^	YbF	2.0174	507.6	2.357	40 904
CCSD(T)^1^	YbF	2.0289	528.2	1.939	41 156
CCSD^21^		2.0127	566.8	3.7885	55 650
RASCI^6^		2.051	529		
CCSD(T)^80^		2.03			38 900
CISD^81^		2.034	502		42 100
DFT^20^		1.987	532		45 000

Exp.^79^					43 260
Exp.^18^		2.0158	506.6674	2.2452	
Exp.^27^			505.5	1.9	
Exp.^78^					43 600
Exp.^30^		2.016514			
Exp.^24^		2.0195	506.616	2.235	

We observe that the extrapolated KRCI bond distances, at about 2.058 Å, are significantly longer (by around 0.04 Å) than experiment,^30^ whereas the coupled cluster calculations show differences from experiment smaller than 0.01 Å, with EOM-CCSD showing slightly larger discrepancies than IHFS-CCSD. Between the extrapolated EOM-CCSD and IHFS-CCSD, we also see small differences between the 4f^14^ and 4f^13^ for EOM-CCSD these differ by around 0.001 Å whereas for IHFS-CCSD the difference is slightly under 0.002 Å, with the 4f^13^ configuration yielding a slightly underestimated value, compared to experiment, something that can be traced back to the differences in model spaces for this configuration.

Our results for harmonic frequencies further indicate that KRCI seems to underestimate the bonding strength in YbF, as the harmonic vibrational frequency is smaller (491 cm^−1^) than experiment (between 505.5 and 506.7 cm^−1^ depending on the experiment). The coupled cluster results, on the other hand, show the typical 5–6 cm^−1^ overestimation of the harmonic frequencies with respect to experiment (something also seen for the anharmonic constants), which can be attributed to lack of triples in the EOM or FS treatment, that would introduce further orbital relaxation. This can be seen in comparison to the unrestricted coupled cluster calculations of Gomes *et al.*,^1^ which in spite of the large value of the *T*_1_ diagnostic, reproduce well the experimental bond lengths, harmonic frequencies and anharmonic constants.

Taken together, our 2-component CCSD-based calculations and the 4-component ones of Gomes *et al.*^1^ compare consistently better to experiment than the other theoretical works for bond lengths, vibrational frequencies and anharmonic constants. For the dissociation energies, on the other hand, the extrapolated calculations presented here do not provide a significant improvement over the results of prior theoretical investigations (quadruple zeta values are closer to the experimental ones for this quantity, see Table 4). Especially, electron attachment values are off, which might be related to the absence of the configuration with a hole in the p orbitals of fluorine.

Moving now to excited states, we start by considering the four lowest excited states, which belong to the 
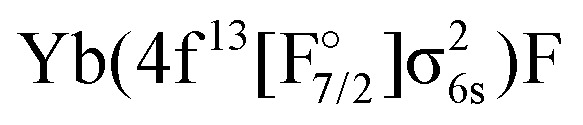
 configuration. These states are well separated from the ground state (the lowest excited state is about 10 000 cm^−1^ above the ground state) and higher excited states. That such states are quite well separated from the ground state would, in our view, tend to exclude the interaction with a low-lying excited state as an explanation for the appearance of the large *T*_1_ diagnostic values observed by Gomes *et al.*^1^ From their spectroscopic constants, presented in Table 6, we see that with the exception of DFT all methods yield similar level splittings of about 500, 1200, and 2000 cm^−1^. To the best of the authors knowledge there is no experimental data available for these states, due to their negligible transition dipole moments for dipole excitations (see for instance Fig. S6 in the ESI†) and small Franck–Condon factors due to the difference in bond distances between these states and the ground state, see Section 3.6.

**Table:** Spectroscopic constants for the lowest excited states 
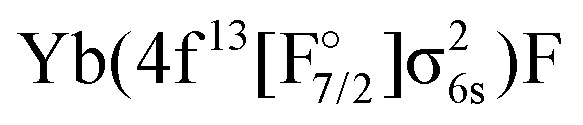
 for different wave function methods using the potential energy curves extrapolated to the basis set limit. In the case of KRCI and MRCI^19^ the ground state is not included in the computation and absolute transition energies are not available. The transition energy (*T*_e_), level splitting (*T*_rel_, energy relative to 2_1/2_), harmonic frequencies (*ω*_e_) and anharmonicity constants (*ω*_e_*χ*_e_) are given in cm^−1^, the equilibrium bond distances (*r*_e_) in Å

State	Method	*T*_e_	*r*_e_	*ω*_e_	*ω*_e_ *χ*_e_	*T*_rel_
2_1/2_	KRCI		1.9200	631	2.51	
	EOM-CCSD	12 568	1.9432	591	2.59	
	IHFS-CCSD	9627	1.9396	599	2.79	
	DFT^20^	3790	1.9570	561		
	MRCI^19^		1.9480	600		
1_3/2_	KRCI		1.9253	628	2.50	540
	EOM-CCSD	13 211	1.9494	588	2.61	643
	IHFS-CCSD	10 180	1.9438	595	2.79	553
	DFT^20^	9520	1.9440	597		5730
	MRCI^19^		1.951 0	598		428
1_5/2_	KRCI		1.9296	622	2.45	1223
	EOM-CCSD	13 703	1.9553	582	2.61	1135
	IHFS-CCSD	10 968	1.9493	589	2.78	1341
	DFT^20^	10 970	1.9360	598		7180
	MRCI^19^		1.9540	594		1021
1_7/2_	KRCI		1.9315	616	2.43	1933
	EOM-CCSD	14 685	1.9556	577	2.62	2117
	IHFS-CCSD	11 645	1.9496	583	2.77	2018
	DFT^20^	16 530	1.936	592		12 740
	MRCI^19^		1.954	589		1709

The smallest equilibrium distance was obtained for the 2_1/2_ state with 1.94 Å for the coupled cluster methods and 0.02 Å less for KRCI. The vibrational frequencies are between 570 and 600 cm^−1^ for the coupled cluster methods and about 30 cm^−1^ higher for KRCI.

For higher excited states, as apparent from the figures in the previous section, the identification and assignment of states gets more difficult and there are differences between the methods. We have nevertheless provided in Table 7 the spectroscopic constants for excited with *Ω* values of 1/2, 3/2, and 5/2, respectively.

**Table:** Spectroscopic constants for selected excited states (complete list presented in Table S17 in the ESI) with *Ω* = 1/2, 3/2, 5/2, starting from 18 000 cm^−1^ for different methods using the values after extrapolation to the basis set limit. Transition energy (*T*_e_), vibrational constant (*ω*_e_), and anharmonicty constant (*ω*_e_*χ*_e_) are given in cm^−1^, the equilibrium bond distance (*r*_e_) in Å. Experimental transitions that were not assigned (n.a.) are also listed. Labels of experimental results are defined in the introduction

*Ω*	Method	State	Configuration	*T*_e_	*r*_e_	*ω*_e_	*ω*_e_ *χ*_e_
1/2	KRCI^a^	3	4f^13^σ^2^_6s_	15 572	1.9038	655	13.57
		5	4f^14^6p^1^	16 189	2.0504	496	2.38
		10	4f^14^5d^1^	19 631	2.0552	490	2.49
	EOM-CCSD	3	4f^14^6p^1^	18 373	2.0004	536	2.72
		4	4f^14^5d^1^	21 448	2.0079	532	2.78
		6	4f^13^σ^2^_6s_	23 241	1.9432	582	4.06
	IHFS-CCSD	3	4f^14^6p^1^	18 249	1.9953	539	2.63
		4	4f^13^σ^2^_6s_	20 267	1.9397	597	2.78
		5	4f^14^5d^1^	21 375	2.0032	533	2.73
	MRCI^19^		4f^13^σ^2^_6s_		1.948	600	
	Exp.^18^ a	3		18106.20		537	3
	Exp.^18^	4	[18.6]_1/2_	18705.06			
	Exp.^24^		[557]	18 574	1.9656	502.15	
	Exp.^24^		[561]	18 699	1.9571		

3/2	KRCI^a^	2	4f^13^σ^2^_6s_	16 206	1.9331	711	8.45
		4	4f^14^6p^1^	17 123	2.0473	499	2.37
		>7	4f^14^5d^1^	24 583	2.0669	470	2.50
	EOM-CCSD	2	4f^14^6p^1^	19 672	1.9971	540	2.72
		4	4f^13^σ^2^_6s_	24 251	1.9537	584	2.64
		5	4f^14^5d^1^	24 468	2.0177	509	2.78
	IHFS-CCSD	2	4f^14^6p^1^	19 543	1.9920	542	2.63
		3	4f^13^σ^2^_6s_	21 222	1.9480	591	2.80
		4	4f^14^5d^1^	24 363	2.0120	512	2.73
	MRCI^19^		4f^13^σ^2^_6s_		1.953	596	
	Exp.^18^	2		19471.49			

5/2	KRCI^a^	3	4f^13^σ^2^_6s_	17 063	1.9302	635	1.41
		>6	4f^14^5d^1^	24 744	2.0639	474	2.48
	EOM-CCSD	2	4f^13^σ^2^_6s_	24 957	1.9536	577	2.62
		3	4f^14^5d^1^	25 023	2.0146	513	2.80
	IHFS-CCSD	2	4f^13^σ^2^_6s_	22 127	1.9499	584	2.77
		3	4f^14^5d^1^	24 919	2.0089	515	2.77
	MRCI^19^		4f^13^σ^2^_6s_		1.954	590	

n.a.	Exp.^26^		[574]	19 150			
	Exp.^26^		[578]	19 280			
	Exp.^27^		C_1_	23035.3		523	2
	Exp.^27^		C_2_	23256.0		507	2
	Exp.^27^		D	26014.8		574.6	2.8

a KRCI transition energies for the 4f^13^ sector were obtained by adding 4144 cm^−1^, an estimate for the energy of the lowest state in this manifold.

The comparison with experimental results allows assignment of the lowest excited state reported in experiments and give some indications for higher states. The lowest *Ω* = 1/2 state observed in experiment can be identified as the 3_1/2_ state (5_1/2_ for KRCI). Spectroscopic parameters agree well with the ones obtained by fitting to the A^2^Π_1/2_ in experiments. A bond distance of 1.9935 Å obtained by fitting to the same states in ref. 34 agrees well with the coupled cluster values for the 3_1/2_ state, the vibrational constant of about 540 cm^−1^ is close to the experimental value of ref. 18. Similarly, the lowest *Ω* = 3/2 state reported by Dunfield *et al.*^18^ can be identified as the 2_3/2_ state (4_3/2_ for KRCI), see Table 7.

The lowest states with *Ω* = 1/2 and *Ω* = 3/2 for this energy range approach asymptotically a state with a Yb(4f^14^5d^1^)F configuration, but if one analyses the EOM-CCSD and IHFS-CCSD orbital composition, significant contributions of the atomic 6p are identified. The *Ω* = 3/2 state is dominated (97%) by a single configuration, corresponding to a HOMO(σ_6s,1/2_) → LUMO+1 where the latter is made up of a mixture of 6p_π_ and 5d_π_ orbitals (the 6p_π_ contributions being the dominant − ≃80% – in the reference YbF^+^ orbitals). The few other significant configurations arise from excitations to higher-lying orbitals with increasingly large (≥50% 5d_π_) contributions. The Π_1/2_ state is also dominated by a single configuration, now corresponding to a HOMO(σ_1/2_) → LUMO transition, and shows a rather similar picture in terms of the relative weights of the 6p_π_ and 5d_π_ orbitals, with very small contributions from the ground-state mixing due to spin–orbit coupling. The splitting in Yb^+^ of ^2^D_3/2_ and ^2^D_5/2_ is 1372 cm^−1^, for ^2^P_1/2_ and ^2^P_3/2_ it is 3330 cm^−1^. The separation between the lowest excited *Ω* = 1/2 states in the closed shell computation is 3779 and 3126 cm^−1^ for EOM-CCSD and IHFS-CCSD, respectively. This is an indication that the two states must be regarded as a fairly strong admixture of 6p and 5d orbitals of *j* = 1/2 or 3/2, as one can expect a much smaller spin–orbit splitting in the axial field of the molecule (about 1/3 of the atomic spin–orbit splitting for the P state). This picture also finds experimental support in recent measurements of hyperfine constants (*d* and *eq*_0*Q*_) for the ground and Π_1/2_ excited state of YbF^9^, where a simple ligand-field model disregarding the contributions from 5d_π_ orbitals predicted values of *d* a factor of 2 larger than the measurements. For bond distances much larger that the equilibrium one the system gets closer to the configurations in Yb^+^ with a dominating 5d contribution.

As already mentioned this energy range above 18 000 cm^−1^ is dense with a large number of excited states that can mix with each other and result in new mixed states, like the [557] and [561] ones.^24^ These will be addressed in Section 3.6.

Uttam *et al.*^27^ reported three unidentified states with energies above 22 000 cm^−1^ which are listed in Table 7 and cannot uniquely be identified with the current results. The one at 26014.8 cm^−1^ has a larger vibrational constant indicating a more strongly bound state, possibly of the 4f^13^σ^2^_6s_ configuration. The vibrational spacing of the two states at 23 000 cm^−1^ rather points to states with a closed f shell.

### 3.6 Perturbation of the 3_1/2_ excited state

Due to the use of different sectors of Fock space to obtain the 4f^14^ and 4f^13^ configurations, the excited states with the same *Ω* values cannot interact among themselves, as is the case within each sector. However, from the discussion above, it is clear that dealing with states which are artificially prevented from interaction makes it difficult to establish a comparison to experiment, for states from about 18 000 cm^−1^ to about 26 000 cm^−1^, which is where these configurations should be the most entangled. In order to remedy that, in the following we introduce a simple adiabatization model (eqn (2)) that allows us to investigate how coupling such states would affect the overall spectra in the aforementioned energy region.

**Fig. 3:**
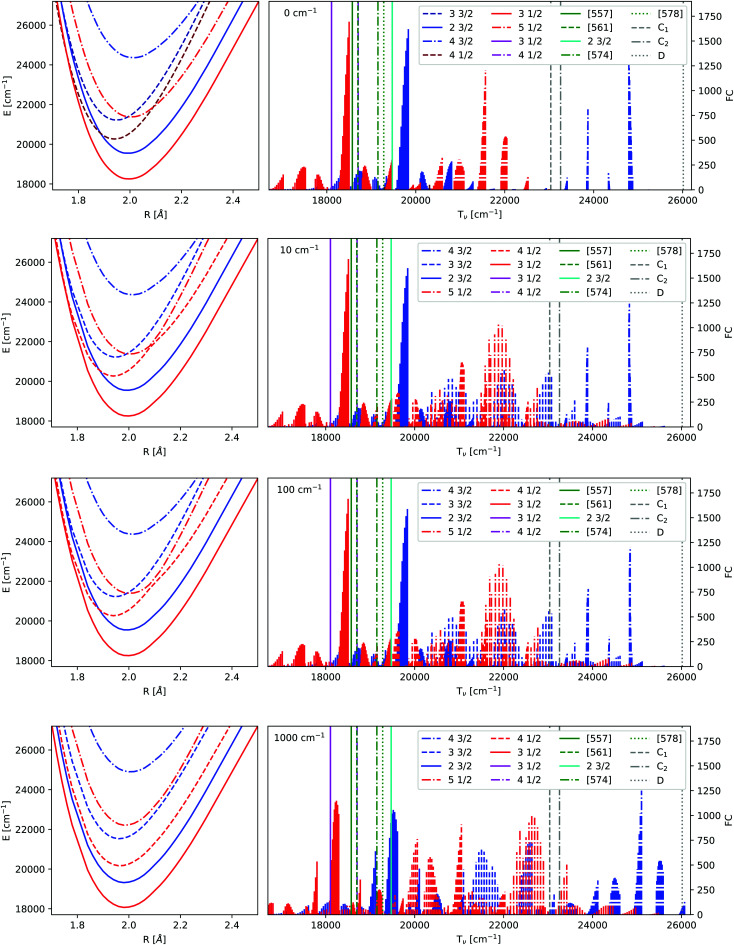
Frank-Condon factors before and after adiabatization for the IHFS-CCSD potential energy curves. C is the coupling strength in Hartree. The lowest 10 vibrational levels of the ground state as well as the lowest 60 vibrational levels of the excited state were computed using the LEVEL program.^82^ The experimental values^18,24,26^ have been added as straight lines, the labels are defined in the introduction and Table 7.

In the following we only consider the IHFS-CCSD potential energy curves, as the spectroscopic parameters are more reliable for CCSD than for KRCI. The coupled cluster results for the two methods are quite similar, and FS-CCSD was selected (because it does not include the (2h,1p) and (1h,2p) transitions with rather large uncertainties). Fig. 3 contains the original FS-CCSD curves as well as the ones obtained after adiabatzation with three different coupling constants. Looking at the potential energy curves for this energetic region, there are two *Ω* = 1/2 and two *Ω* = 3/2 states of Yb(4f^14^6p^1^)F and Yb(4f^14^5d^1^)F configurations originating from the (0h,1p) sector. For both *Ω* values there is an additional state with a 
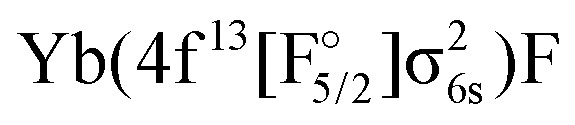
 configuration stemming from the (1h,0p) sector. By looking at the KRCI results one expects additional states belonging to the 
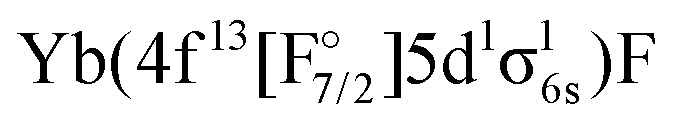
 configuration for this energy range, which will not be included in the current considerations.

As already mentioned earlier the lowest *Ω* = 1/2 and *Ω* = 3/2 states can be identified clearly and assigned to experimental observations. There are several experimental states in this energy region attributed to the mixing of states. The [557] and [561] ones^24^ are assumed to arise from a mixing of the 3_1/2_ and 4_1/2_. The vibrational constant of the perturbing state (4_1/2_) was estimated to be 605 cm^−1^ in ref. 18. This agrees with the 4_1/2_ state in Fig. 3 with a 
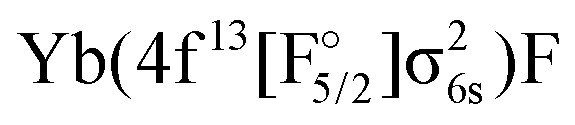
 configuration, see also Table 7. [574] and [578]^26^ have not been identified and since their *Ω* value is unknown, we were not able to assign them to a configuration.

Next we take a look at the changes introduced by adiabatization. For small and intermediate coupling strengths there are no major differences in the potential energy curves, although close to the crossing points the potentials are deformed. Intermediate coupling strengths with slightly deformed potentials close to the avoided crossings will be the most realistic description. For very large coupling strengths one obtains parallel potential energy curves due to the strong repulsion. This also results in a major change of the spectra above 19 000 cm^−1^. One of the differences between the adiabatic spectrum and the upermost one in Fig. 3 is that the Frank-Condon factors of the 4_1/2_ state, which is of the Yb(4f^13^σ^2^_6s_)F configuration, are now noticeable and the spacing of the energy levels of the 5_1/2_ is changed. Similarly, transitions belonging to the 5_1/2_ appear.

The influence of adiabatization on spectroscopic parameters can be investigated by comparing spectroscopic constants calculated for the IHFS-CCSD curves without and with a coupling of 100 cm^−1^ (Table 8). We observe that for this coupling strength, there are small but non-negligible changes for the excitation energies, harmonic frequencies and anharmonicity constants, for all but the fourth and fifth *Ω* = 1/2 states; there, the coupling does seem to significantly change the anharmonicity constants. Equilibrium distances, on the other hand, are largely unperturbed in all cases. Furthermore, as expected from the preceding discussion, no changes are observed for the ground-state, since it is too separated in energy from the other electronic states.

**Table:** Spectroscopic data obtained by fitting Morse potentials to the lowest points of the potential energy curves obtained with FSCC for the extrapolated basis set (CBS). This table combines results from both sectors starting either with a closed (f^14^) or open (f^13^) f-shell. Additionally, the table contains spectroscopic parameters after adiabatization with a specific coupling constant (C). The transition energy (*T*_e_), vibrational constant (*ω*_e_), and anharmonicty constant (*ω*_e_*χ*_e_) are given in cm^−1^, the equilibrium bond distance (*r*_e_) in Å

*Ω*	CBS					*C* = 100 cm^−1^				
	State	*r*_e_	*ω*_e_	*ω*_e_ *χ*_e_	*T*_e_	State	*r*_e_	*ω*_e_	*ω*_e_ *χ*_e_	*T*_e_
1/2	f^14^ – 1	2.018	515	2.9	0	1	2.018	515	2.8	0
	f^13^ – 2	1.940	599	2.8	9627	2	1.940	599	2.8	9617
	f^14^ – 2	1.995	539	2.6	18 249	3	1.995	538	2.6	18 247
	f^13^ – 3	1.940	597	2.8	20 267	4	1.935	603	8.6	20 258
	f^14^ – 3	2.003	533	2.7	21 375	5	2.002	586	0.4	21 359
	f^14^ – 4	1.964	581	1.8	31 416	6	1.964	581	1.8	31 419

3/2	f^13^ – 1	1.944	595	2.8	10 180	1	1.944	594	2.8	10 170
	f^14^ – 1	1.992	542	2.6	19 543	2	1.992	542	2.6	19 540
	f^13^ – 2	1.948	591	2.8	21 222	3	1.948	591	2.8	21 217
	f^14^ – 2	2.012	512	2.7	24 363	4	2.012	512	2.7	24 369

5/2	f^13^ – 1	1.949	589	2.8	10 967	1	1.949	589	2.8	10 960
	f^13^ – 2	1.950	584	2.8	22 127	2	1.950	583	2.8	22 117
	f^14^ – 1	2.009	515	2.8	24 919	3	2.009	516	2.7	24 926

## 4 Conclusion

In this manuscript we have presented a study of the ground and excited states of the YbF molecule, with 2-component multireference CI, equation-of-motion and Fock space coupled cluster approaches (in all cases, performing extrapolations to the complete basis set limit). In particular, we have focused on obtaining electronic states up to around 24 000 cm^−1^ arising from configurations which differ in the occupation of the 4f shell (4f^14^ and 4f^13^), which are very difficult to treat on the same footing due to a number of subtle correlation and relaxation effects.

In order to achieve such a balanced description, our strategy consisted of starting from YbF^+^ and YbF^−^, in order to arrive at the wavefunctions for YbF through the (1h,0p) and (0h,1p) sectors of Fock space. Once obtained, electronic states with same *Ω* values coming from these different sectors are further coupled through a simple adiabatization model in which the coupling strength is taken as a constant.

As a general rule we find that the CI calculations do capture the essential physics of the system, though they are not as reliable as the coupled cluster approaches for excitation energies, bond lengths, harmonic vibrational frequencies and anharmonic constants. In effect, the coupled cluster calculations for the (1h,0p) and (0h,1p) sectors yield the same potential energy curves for the ground state, for internuclear distances up to around 2.8 Å, which is sufficient to capture the bound regions of all states under consideration,

We have determined that the lowest lying excited states arise from the 
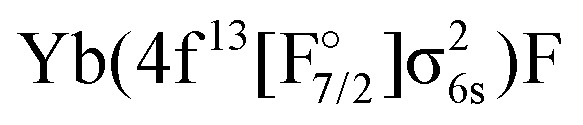
 configuration, with transition energies of around 10 000 cm^−1^, and a splitting about 2000 cm^−1^. These states are, however, not generally accessible in experiment due to their low dipolar intensity and significantly shifted minima of the potential energy curve resulting in small Frank-Condon factors.

The next set of states, coming above 18 000 cm^−1^, arise from the Yb(4f^14^6p^1^)F, Yb(4f^14^5d^1^)F, 
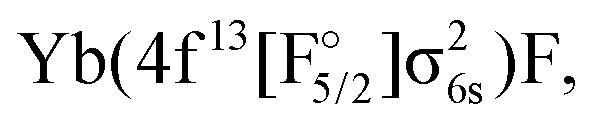
 and 
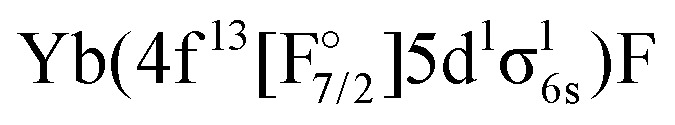
 configurations. Among these, the 
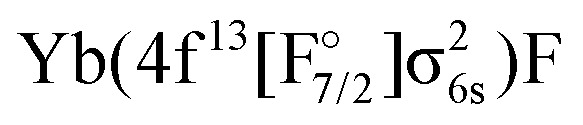
 configurations generally display the shortest equilibrium distances and deepest potential well, while the Yb(4f^14^5d^1^)F and Yb(4f^14^6p^1^)F configurations exhibits the largest bond distances and smallest harmonic frequencies, with the other configurations falling somewhere in between. The lowest *Ω* = 1/2 and *Ω* = 3/2 states of this group show a Yb(4f^14^6p^1^)F orbital composition around the ground-state equilibrium structure, though for longer bond lengths they asymptotically approach the Yb(4f^14^5d^1^)F configuration.

We note that configurations with three unpaired electrons, such as 
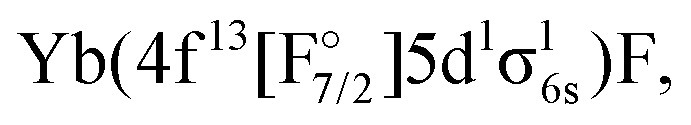
 were only considered with the KRCI method, which has larger uncertainties. This only allows us to make some qualitative statements, *e.g.* that their bond distances and vibrational constant should be between the values for the other configurations and that they should be higher in energy than the lowes excited Yb(4f^14^6p^1^)F and 
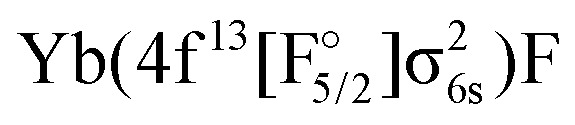
 states.

A simple method was applied in order to adiabatize the curves obtained for different sectors and reference wave functions. It was applied to potential energy curves between 18 000 and 26 000 cm^−1^ and small changes of the Franck–Condon factors were observed. The influence on spectroscopic constant was minor, with the exception of the asymmetry constant for two states. However, the approximation introduced (same coupling strength for all states and all geometries) is perhaps not flexible enough, and more sophisticated models should be investigated.

## Conflicts of interest

There are no conflicts to declare.

## Supplementary Material

CP-023-D1CP03701C-s001
