# Supplementary material for: Electronic spectra of ytterbium fluoride from relativistic electronic structure calculations
Source: Phys Chem Chem Phys. 2021 Oct 1;23(39):22330–43. doi: 10.1039/d1cp03701c (PMC8514048; doi:10.1039/d1cp03701c)
Supplement: CP-023-D1CP03701C-s001 [file CP-023-D1CP03701C-s001.pdf]

Electronic Supplementary Material (ESI) for Physical Chemistry Chemical Physics.
This journal is © the Owner Societies 2021

Supplementary Information: Electronic Spectra of Ytterbium Fluoride from Relativistic Electronic Structure Calculations

Johann V. Pototschnig,*,† Kenneth G. Dyall,*,‡ Lucas Visscher,*,† and André Severo Pereira Gomes*,¶

†*Amsterdam Center for Multiscale Modeling, Department of Theoretical Chemistry, Faculty of Sciences, VU University Amsterdam, De Boelelaan 1083, NL-1081 HV Amsterdam, The Netherlands*

‡*Dirac Solutions, 10527 NW Lost Park Drive, Portland, OR 97229, U.S.A.*

¶*Université de Lille, CNRS, UMR 8523 – PhLAM – Physique des Lasers, Atomes et Molécules, F-59000 Lille, France*

E-mail: j.v.pototschnig@vu.nl; diracsolutions@gmail.com; l.visscher@vu.nl; andre.gomes@univ-lille.fr

This is the supplementary information for the manuscript entitled: "Electronic Spectra of Ytterbium Fluoride from Relativistic Electronic Structure Calculations" by J. V. Pototschnig, K. G. Dyall, L. Visscher and A. S. P. Gomes.

In section 1 a closer look is taken at the orbitals and their energies for the two different references, either with a closed or open f-shell. Orbital energies for different diatomic distances are depicted alongside orbitals for the YbF diatomic. Section 2 contains additional results obtained for Kramers restricted configuration interaction. Firstly, we compare results obtained with the X2C-AMFI code to full 4-component computations. Next, poten-

tial energy curves, including spectroscopic parameters, and dipole moments–both transition dipole moments (TDM) and permanent electric dipole moments (PEDM)–for different basis set sizes are presented. Subsequently, the problem of the relative position of the f^{13} and f^{14} states is addressed and its basis set dependence. Lastly, transition dipole moments are listed. Section 3 contains the potential energy curves and spectroscopic parameters for different basis set sizes applying the Fock space coupled cluster method and spectroscopic parameters for the states after adiabatic mixing as presented in the main text. An extended list of equation-of-motion coupled cluster transition energies for the Yb^+ is listed in section 4. This section also contains EOM-CCSD potential energy curves for different basis set sizes. Finally, in section 6 Franck-Condon factors for potentials stemming from the different methods are depicted and compared. Additional figures (orbital pictures, basis set convergence) can be found in the first version of this manuscript on arXiv.[1]

1 Orbitals

1.1 Orbital energies

In this part the orbital energies obtained by AOC-SCF for several internuclear separations are shown.

YbF⁻

YbF

YbF⁺

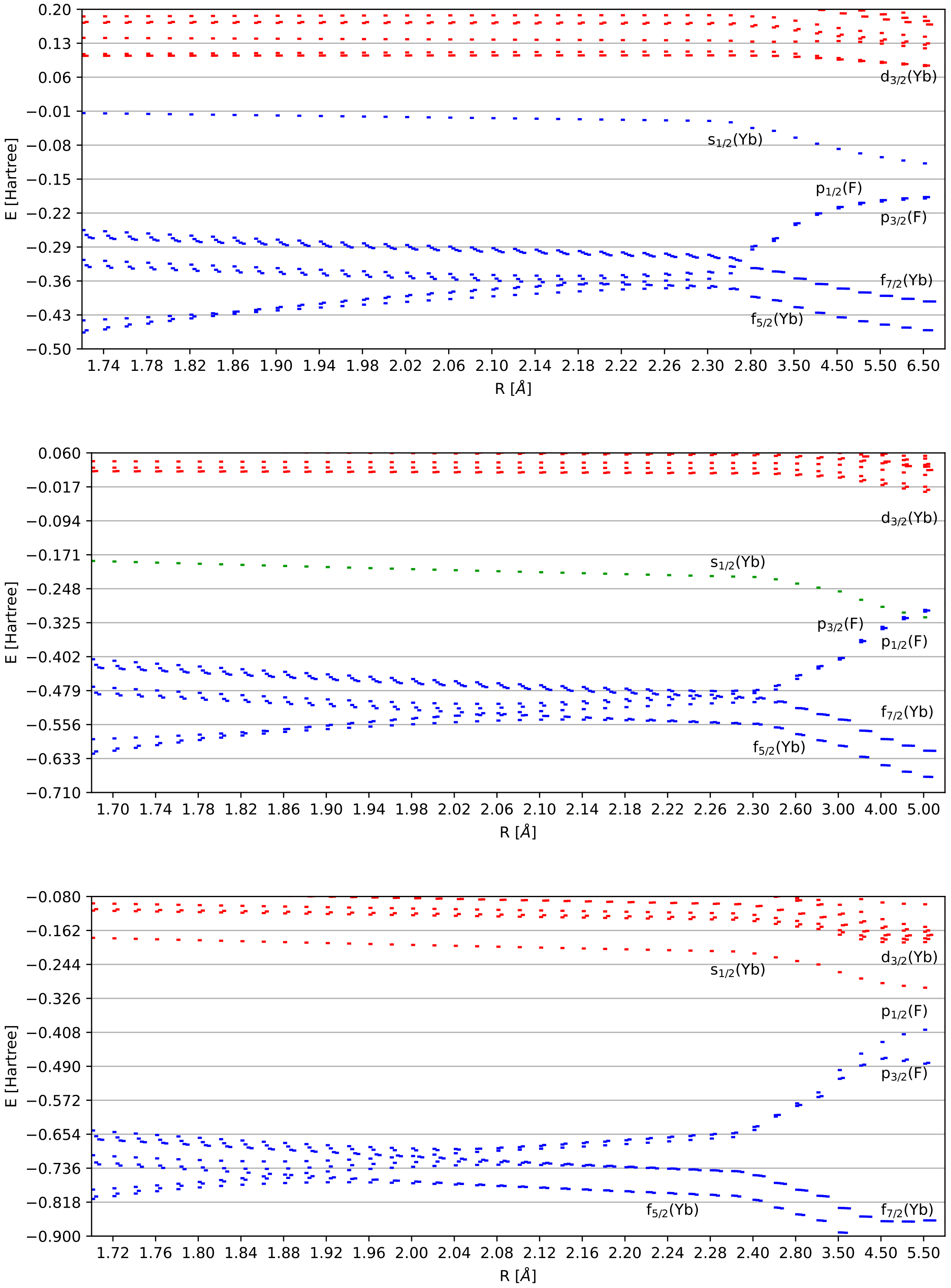

Figure S1: Orbital energies for different bond distances. Blue, green, and red are occupied, partially filled, and virtual orbitals, respectively.

1.2 Diatomic orbitals - equilibrium distance

This section contains orbitals of the YbF diatomic for a internuclear distance close to the ground state equilibrium one.

Table S1: Orbitals obtained from an AOC-SCF computations either with a fully occupied (f^{14}) or single hole (f^{13}) f-shell for YbF with a distance of 2 Å.For each orbital we list the orbital energy according to a Koopman's definition.[2,3] The contributing atomic orbitals contributing have been given in the first column.

designation	f^{14}	f^{13}
	-2.475129022	-2.606565796
25<br>$5s_{1/2}$(Yb)	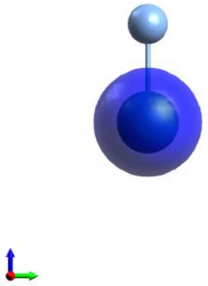	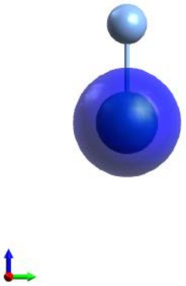
	-1.511889433	-1.611193447
26<br>$5p_{1/2}$(Yb)<br>$2s_{1/2}$(F)	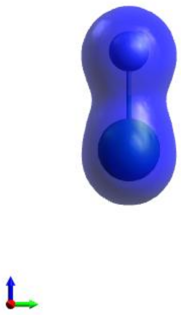	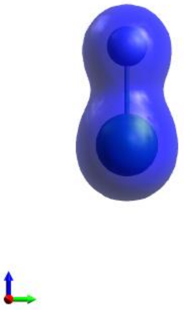

Continued on next page

Table S1 – *Continued from previous page*

designation	f^{14}	f^{13}
	-1.435750686	-1.539604687
27<br>$5p_{1/2}$(Yb)<br>$2s_{1/2}$(F)	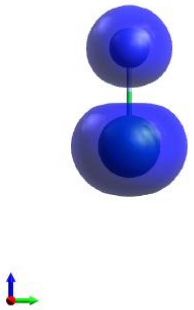	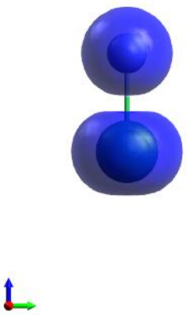
	-1.229656975	-1.323836039
28<br>$5p_{3/2}$(Yb)	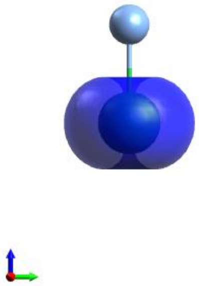	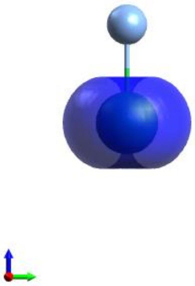

Continued on next page

Table S1 – *Continued from previous page*

designation	f^{14}	f^{13}
29<br>$5p_{3/2}$(Yb)	-1.210630046<br>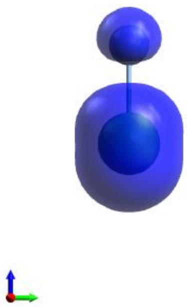	-1.311522305<br>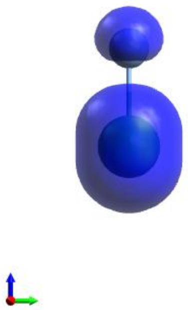
30<br>$4f_{5/2,7/2}$(Yb)<br>$2p_{1/2,3/2}$(F)	-0.594742161<br>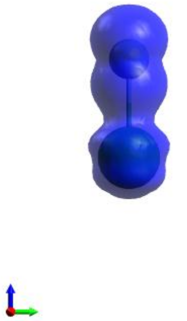	-0.774346397<br>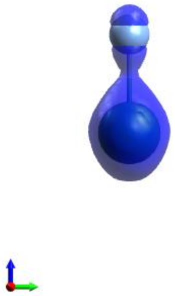

Continued on next page

Table S1 – *Continued from previous page*

designation	f^{14}	f^{13}
	-0.584034308	-0.773626615
31<br>$4f_{5/2,7/2}$(Yb)<br>$2p_{1/2,3/2}$(F)	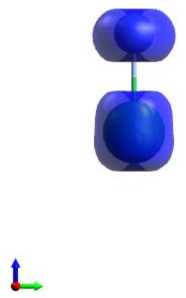	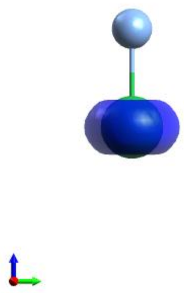
	-0.582311174	-0.772523010
32<br>$4f_{5/2,7/2}$(Yb)<br>$2p_{1/2,3/2}$(F)	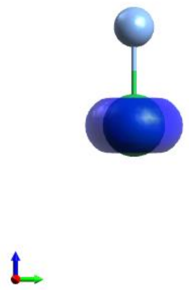	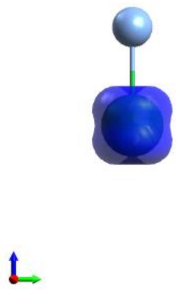

Continued on next page

Table S1 – *Continued from previous page*

designation	f^{14}	f^{13}
	-0.574792083	-0.719689709
33<br>$4f_{5/2,7/2}$(Yb)<br>$2p_{1/2,3/2}$(F)	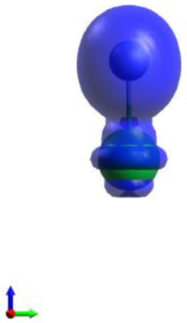	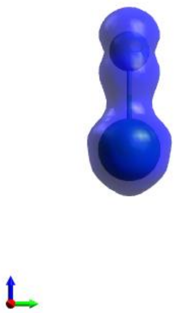
	-0.561976545	-0.715455417
34<br>$4f_{5/2,7/2}$(Yb)<br>$2p_{1/2,3/2}$(F)	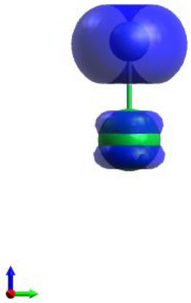	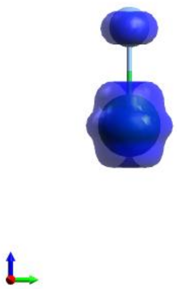

Continued on next page

Table S1 – *Continued from previous page*

designation	f^{14}	f^{13}
	-0.55211333	-0.715038003
35<br>$4f_{5/2,7/2}$(Yb)<br>$2p_{1/2,3/2}$(F)	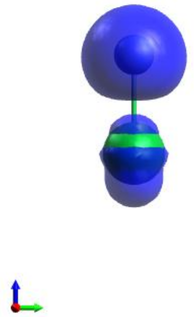	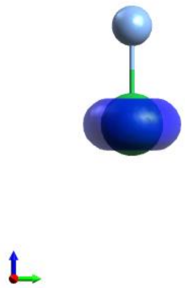
	-0.523207455	-0.713718461
36<br>$4f_{5/2,7/2}$(Yb)<br>$2p_{1/2,3/2}$(F)	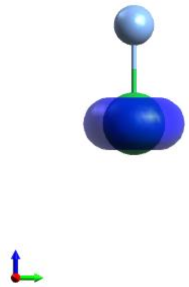	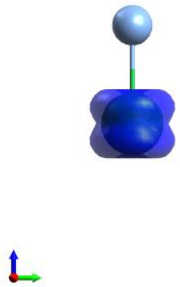

Continued on next page

Table S1 – *Continued from previous page*

designation	f^{14}	f^{13}
	-0.521323830	-0.653507491
37<br>$4f_{5/2,7/2}$(Yb)<br>$2p_{1/2,3/2}$(F)	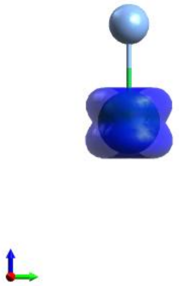	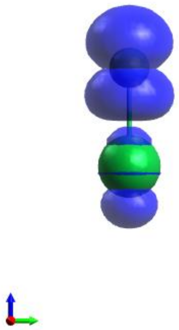
	-0.515413462	-0.644769626
38<br>$4f_{5/2,7/2}$(Yb)<br>$2p_{1/2,3/2}$(F)	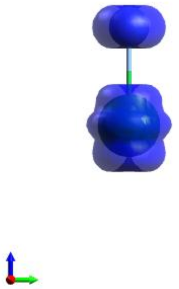	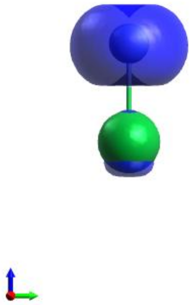

Continued on next page

Table S1 – *Continued from previous page*

designation	f^{14}	f^{13}
	-0.509924741	-0.639112727
39<br>$4f_{5/2,7/2}$(Yb)<br>$2p_{1/2,3/2}$(F)	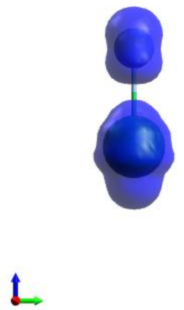	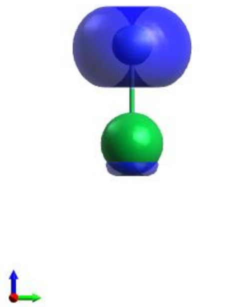
	-0.129066590	-0.241206568
40<br>$4f_{5/2,7/2}$(Yb)<br>$2p_{1/2,3/2}$(F)	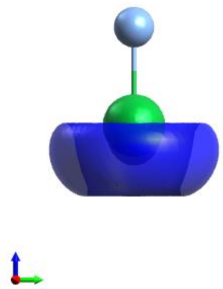	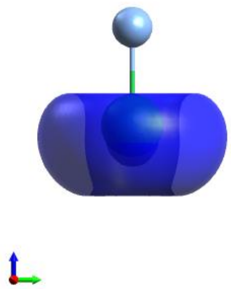

2 Kramers restricted configuration interaction (KRCI)

2.1 Hamiltonian

Due to limitations in the implementation it is not possible to run the more accurate $^2DC^M$ [4] in combination with Kramers-restricted configuration interaction. Therefore, we have run the computations with the X2C-AMFI[5,6] Hamiltonian. In order to estimate the influence of this approximation we compare the results with the 4-component Dirac-Coulomb-Breit Hamiltonian (DCB)[7] in tables S2 and S3 for the Yb cation. The effect of the basis set

Table S2: Transition energies (cm^{-1}) and line strengths / squared transition dipole moments ($e^2a_0^2$) for the Yb cation. Reference values have been obtained from the NIST database,[8] the computed values were obtained for different basis sets with Kramers-restricted configuration interaction.

state	conf	NIST[9]	2z (x2c)	3z (x2c)	4z (x2c)	2z (DCB[7])	3z (DCB[7])	4z (DCB[7])
		E	E	E	E	E	E	E
$^2S_{1/2}$	4f^{14}6s	0	0	0	0	0	0	0
$^2D_{3/2}$	4f^{14}5d	22961	23322	22802	23606	23389	22871	23674
$^2D_{5/2}$	4f^{14}5d	24333	23882	23321	24117	23945	23386	24182
$^2P^\circ_{1/2}$	4f^{14}6p	27062	25210	24533	25331	25241	24564	25362
$^2P^\circ_{3/2}$	4f^{14}6p	30392	28104	27385	28153	28139	27422	28189
		S	TDM^2	TDM^2	TDM^2	TDM^2	TDM^2	TDM^2
$^2D_{3/2}$	4f^{14}5d		0.0	0.0	0.0	0.0	0.0	0.0
$^2D_{5/2}$	4f^{14}5d		0.0	0.0	0.0	0.0	0.0	0.0
$^2P^\circ_{1/2}$	4f^{14}6p	6.1	5.3	5.5	5.3.	5.3	5.5	5.3
$^2P^\circ_{3/2}$	4f^{14}6p	11.4	16.4	17.3	16.6	16.4	17.3	16.6

Table S3: Transition energies between states with an open f-shell obtained with configuration interaction. Transition energies relative to the $^2F^\circ_{7/2}$ state are listed.

			x2c			DCB[7]		
state	conf	NIST[9]	2z	3z	4z	2z	3z	4z
$^2F^\circ_{7/2}$	4f$^{13}\sigma^2_{6s}$	0	0	0	0	0	0	0
$^3\,[3/2]^\circ_{5/2}$	4f^{13}5d 6s	5340	4260	5538	4618	4349	5630	4711
$^3\,[3/2]^\circ_{3/2}$	4f^{13}5d 6s	7339	6387	7822	7123	6478	7917	7218
$^3\,[11/2]^\circ_{9/2}$	4f^{13}5d 6s	8806	8214	9325	8314	8304	9419	8408
$^3\,[11/2]^\circ_{11/2}$	4f^{13}5d 6s	9144	8320	9431	8447	8409	9524	8540

is much larger and since no significant impact can be expected we used the X2C-AMFI Hamiltonian to obtain the data.

2.2 Closed f-shell - potential energy curves

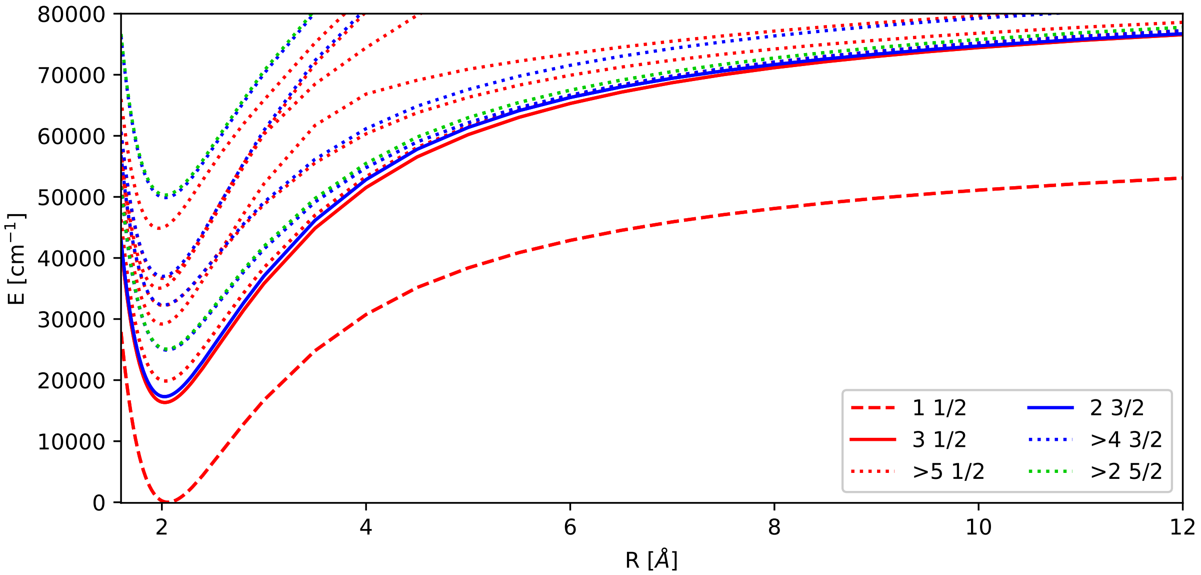

Figure S2: KRCI PECs for states with f^{14} for the quadruple zeta basis sets.

Table S4: Spectroscopic constants for the different electronic states ($\Omega = 1/2, 3/2, 5/2$) with f^{14} obtained by KRCI for the double and triple zeta basis sets. Vibrational constant (ω_e), anharmonicty constant ($\omega_e\chi_e$), and transition energy (T_e), are given in cm^{-1}, the equilibrium bond distance (r_e) in Å.

Ω	state	v2z				v3z			
		r_e	ω_e	$\omega_e\chi_e$	T_e	r_e	ω_e	$\omega_e\chi_e$	T_e
1/2	1	2.0609	493	2.29	0	2.0594	493	2.27	0
1/2	2	2.0316	520	2.30	17319	2.0299	521	2.27	16663
1/2	3	2.0397	513	2.49	21252	2.0382	513	2.33	20270
1/2	4	2.1095	444	2.25	35868	2.0079	547	2.16	30993
1/2	5	2.0440	503	1.88	37977	2.0531	481	2.51	33700
1/2	6	2.0080	574	2.83	42795	1.9871	579	2.31	37776
1/2	7	2.0608	509	2.01	52474	2.0149	537	1.98	42915
3/2	1	2.0281	524	2.32	18425	2.0266	525	2.28	17667
3/2	2	2.0779	472	2.31	26817	2.0592	486	2.34	25450
3/2	3	2.1120	448	2.27	36017	2.0545	483	2.50	33792
3/2	4	2.0558	509	2.03	54498	2.0114	538	1.98	43720
5/2	1	2.0761	474	2.31	27016	2.0575	488	2.33	25572

Table S5: Spectroscopic constants for the different electronic states ($\Omega = 1/2, 3/2, 5/2$) with f^{14} obtained by KRCI for the quadruple zeta and extrapolation to the complete basis set. Vibrational constant (ω_e), anharmonicty constant ($\omega_e\chi_e$), and transition energy (T_e), are given in cm^{-1}, the equilibrium bond distance (r_e) in Å.

Ω	state	v4z				CBS			
		r_e	ω_e	$\omega_e\chi_e$	T_e	r_e	ω_e	$\omega_e\chi_e$	T_e
1/2	1	2.0587	492	2.30	0	2.0829	465	2.40	0
1/2	2	2.0290	521	2.30	16338	2.0504	496	2.38	16189
1/2	3	2.0351	515	2.37	19857	2.0552	490	2.49	19631
1/2	4	2.0012	554	2.23	29183	2.0154	537	2.31	28043
1/2	5	2.0169	509	2.97	32206	2.0131	505	3.44	31208
1/2	6	1.9727	587	2.35	35042	1.9792	570	2.41	33312
1/2	7	2.0103	542	1.87	36658	2.0269	525	1.79	32245
3/2	1	2.0260	524	2.31	17296	2.0473	499	2.37	17123
3/2	2	2.0496	492	2.40	24927	2.0669	470	2.50	24583
3/2	3	2.0193	509	2.86	32297	2.0160	503	3.22	31295
3/2	4	2.0058	546	1.98	36990	2.0215	530	1.96	32245
5/2	1	2.0474	495	2.39	25067	2.0639	474	2.48	24744

2.3 Closed f-shell - dipole moments

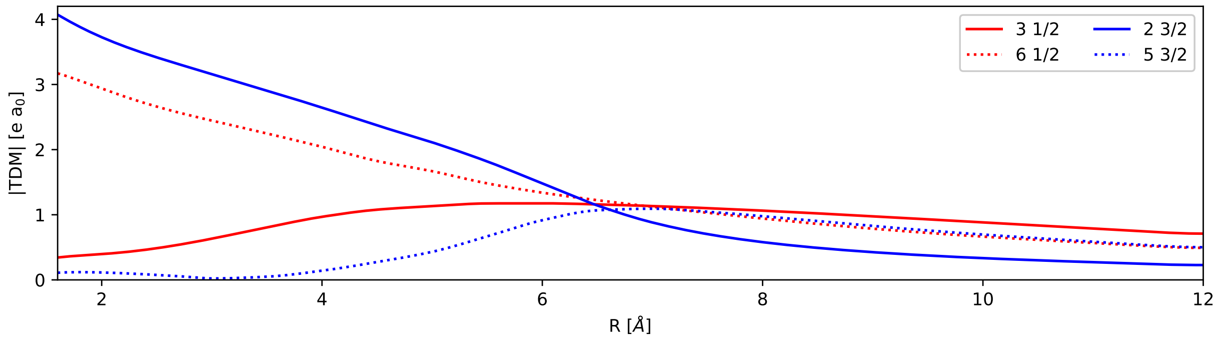

Figure S3: KRCI TDMs for states with f^{14} for the quadruple zeta basis sets.

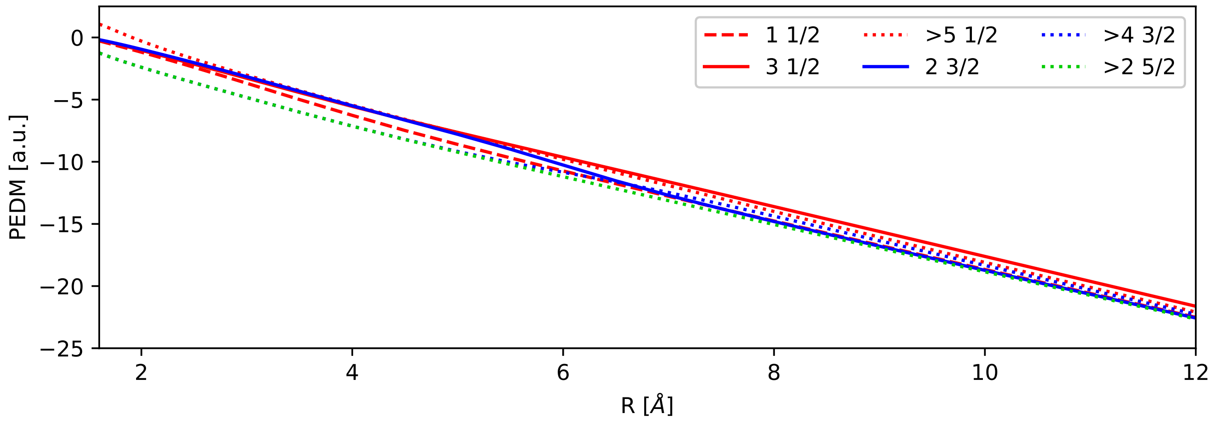

Figure S4: KRCI PEDMs for states with f^{14} for the quadruple zeta basis sets.

2.4 Open f-shell - potential energy curves

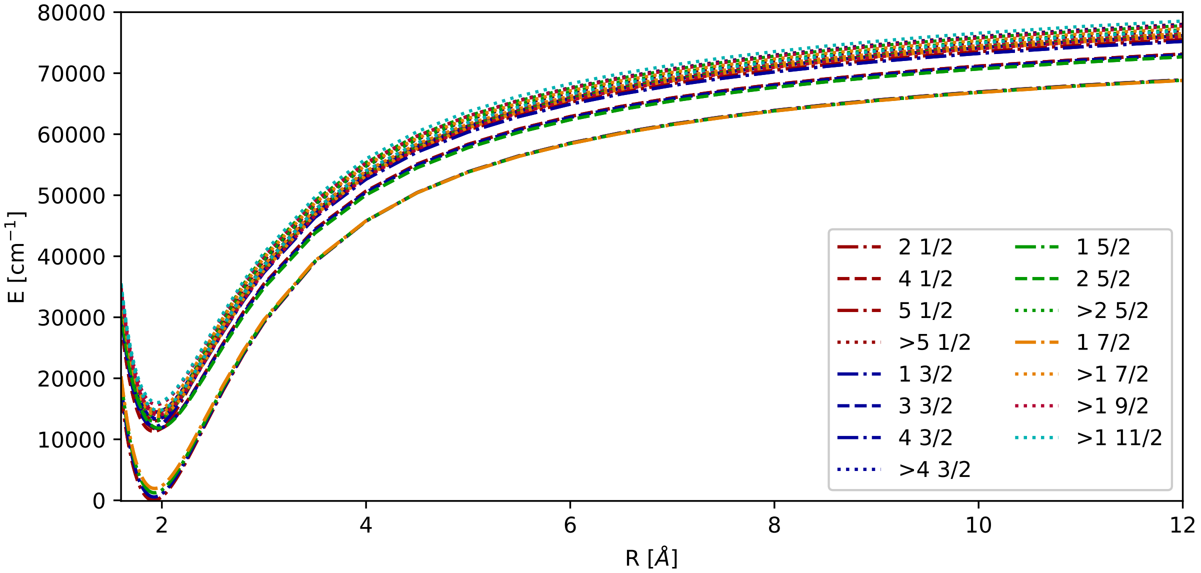

Figure S5: KRCI PECs for states with f^{13} for the triple zeta basis sets.

Table S6: Spectroscopic constants for the different electronic states ($\Omega = 1/2, 3/2, 5/2$) with f^{13} obtained by KRCI for the double and triple zeta basis sets. Vibrational constant (ω_e), anharmonicty constant ($\omega_e\chi_e$), and transition energy (T_e), are given in cm^{-1}, the equilibrium bond distance (r_e) in Å.

Ω	state	v2z				v3z			
		r_e	ω_e	$\omega_e\chi_e$	T_e	r_e	ω_e	$\omega_e\chi_e$	T_e
1/2	1	1.9254	627	2.53	0	1.9188	631	2.73	0
1/2	2	1.9762	510	0.23	10834	1.9540	517	1.24	11083
1/2	3	1.9321	702	7.89	11243	1.9314	684	4.10	11352
1/2	4	1.9568	558	2.79	12833	1.9404	584	3.07	12811
1/2	5	1.9555	575	2.64	13071	1.9469	582	2.73	12946
1/2	6	1.9689	578	2.38	13663	1.9631	569	2.12	13947
1/2	7	1.9645	578	2.25	13884	1.9566	580	2.61	14069
3/2	1	1.9305	624	2.54	557	1.9239	628	2.76	545
3/2	2	1.9797	556	2.43	10849	1.9677	561	2.48	11212
3/2	3	1.9331	597	5.44	12028	1.9262	625	3.62	12045
3/2	4	1.9706	587	0.62	12938	1.9527	576	2.65	13073
3/2	5	1.9639	575	1.88	13087	1.9519	569	2.22	13194
3/2	6	1.9621	576	2.73	13632	1.9596	576	2.24	13797
3/2	7	1.9592	591	2.88	13966	1.9568	594	2.69	14083
5/2	1	1.9341	619	2.54	1272	1.9277	623	2.77	1227
5/2	2	1.9837	554	2.44	10843	1.9736	559	2.49	11295
5/2	3	1.9420	548	5.37	12945	1.9293	580	6.14	12923
5/2	4	1.9568	625	3.55	13211	1.9563	622	0.70	13264
5/2	5	1.9664	593	0.60	13543	1.9553	580	1.95	13670
5/2	6	1.9558	578	2.67	14218	1.9474	588	2.81	14225
7/2	1	1.9355	613	2.50	2017	1.9290	617	2.75	1931
7/2	2	1.9753	566	2.46	13192	1.9665	570	2.45	13424
7/2	3	1.9712	569	2.47	13565	1.9613	573	2.48	13819
7/2	4	1.9626	569	2.41	14333	1.9458	585	2.94	14475
9/2	1	1.9776	563	2.48	13226	1.9707	568	2.47	13576
9/2	2	1.9755	566	2.48	13544	1.9665	571	2.50	13933
9/2	3	1.9709	571	2.51	14319	1.9592	577	2.54	14880
11/2	1	1.9769	564	2.48	13466	1.9691	569	2.49	14001
11/2	2	1.9744	568	2.49	14177	1.9628	576	2.52	14904

Table S7: Spectroscopic constants for the different electronic states ($\Omega = 1/2, 3/2, 5/2$) with f^{13} obtained by KRCI for the quadruple zeta and extrapolation to the complete basis sets. Vibrational constant (ω_e), anharmonicty constant ($\omega_e\chi_e$), and transition energy (T_e), are given in cm^{-1}, the equilibrium bond distance (r_e) in Å.

Ω	state	v4z				CBS			
		r_e	ω_e	$\omega_e\chi_e$	T_e	r_e	ω_e	$\omega_e\chi_e$	T_e
1/2	1	1.9183	632	2.60	0	1.9200	631	2.51	0
1/2	2	1.9191	583	8.23	11329	1.9038	655	13.57	11428
1/2	3	1.9497	641	0.17	11672	1.9603	620	0.08	11876
1/2	4	1.9366	593	2.73	13044	1.9360	598	2.50	13206
1/2	5	1.9426	588	2.65	13240	1.9417	592	2.56	13443
1/2	6	1.9566	576	2.40	14295	1.9539	582	2.71	14531
1/2	7	1.9503	580	2.75	14504	1.9479	578	2.71	14808
3/2	1	1.9236	628	2.61	543	1.9253	628	2.50	540
3/2	2	1.9589	530	0.82	11810	1.9505	516	0.80	12202
3/2	3	1.9306	667	4.87	12069	1.9331	711	8.45	12062
3/2	4	1.9483	582	2.55	13356	1.9473	585	2.47	13549
3/2	5	1.9440	578	2.90	13495	1.9408	584	3.30	13699
3/2	6	1.9545	575	2.67	14251	1.9528	574	2.97	14567
3/2	7	1.9575	591	1.96	14429	1.9601	589	1.50	14667
5/2	1	1.9276	623	2.61	1227	1.9296	622	2.46	1223
5/2	2	1.9690	563	2.45	11828	1.9677	564	2.42	12196
5/2	3	1.9282	614	3.10	12923	1.9302	635	1.41	12919
5/2	4	1.9579	592	1.08	13556	1.9627	571	0.93	13747
5/2	5	1.9482	582	2.57	14030	1.9451	584	3.07	14278
5/2	6	1.9449	593	2.66	14442	1.9453	596	2.53	14590
7/2	1	1.9293	617	2.59	1935	1.9315	616	2.43	1933
7/2	2	1.9627	573	2.43	13764	1.9620	574	2.42	13994
7/2	3	1.9552	576	2.39	14284	1.9528	577	2.31	14607
7/2	4	1.9424	595	2.81	14729	1.9422	601	2.69	14905
9/2	1	1.9669	570	2.49	13962	1.9663	571	2.49	14224
9/2	2	1.9613	575	2.48	14516	1.9596	576	2.45	14923
9/2	3	1.9494	584	2.59	15691	1.9444	588	2.62	16262
11/2	1	1.9645	572	2.49	14706	1.9633	574	2.48	15202
11/2	2	1.9525	584	2.52	16023	1.9472	588	2.51	16817

2.5 Open f-shell - dipole moments

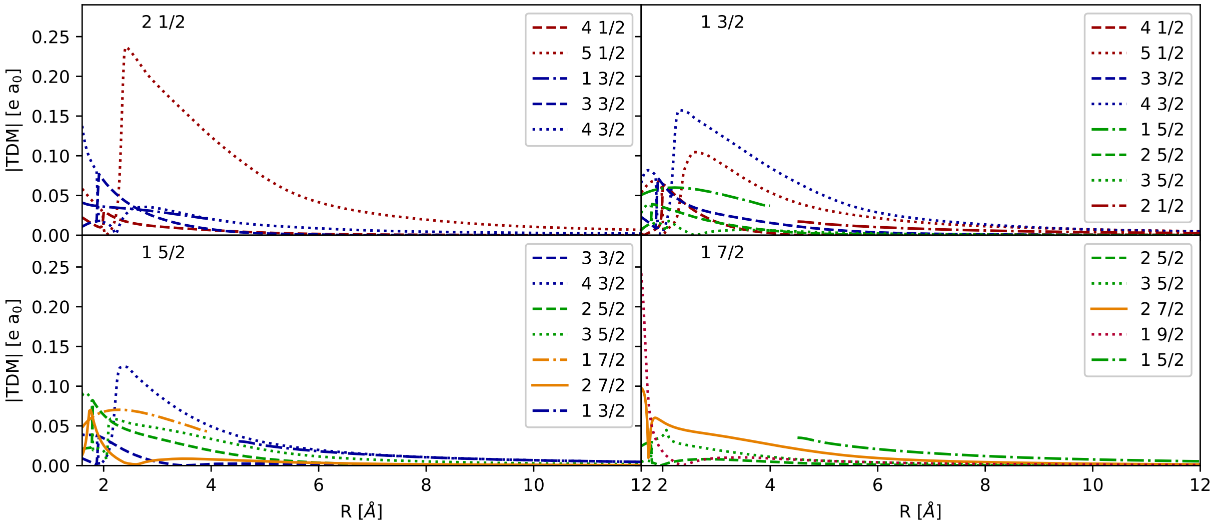

Figure S6: KRCI TDMs for states with f[14] for the quadruple zeta basis sets.

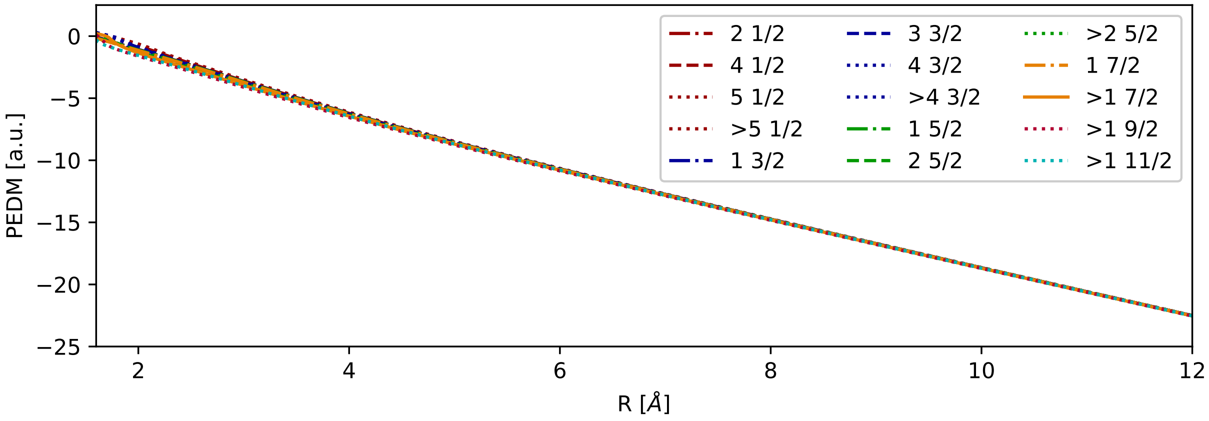

Figure S7: KRCI PEDMs for states with f[13] for the quadruple zeta basis sets.

2.6 Combined potentials, shifting

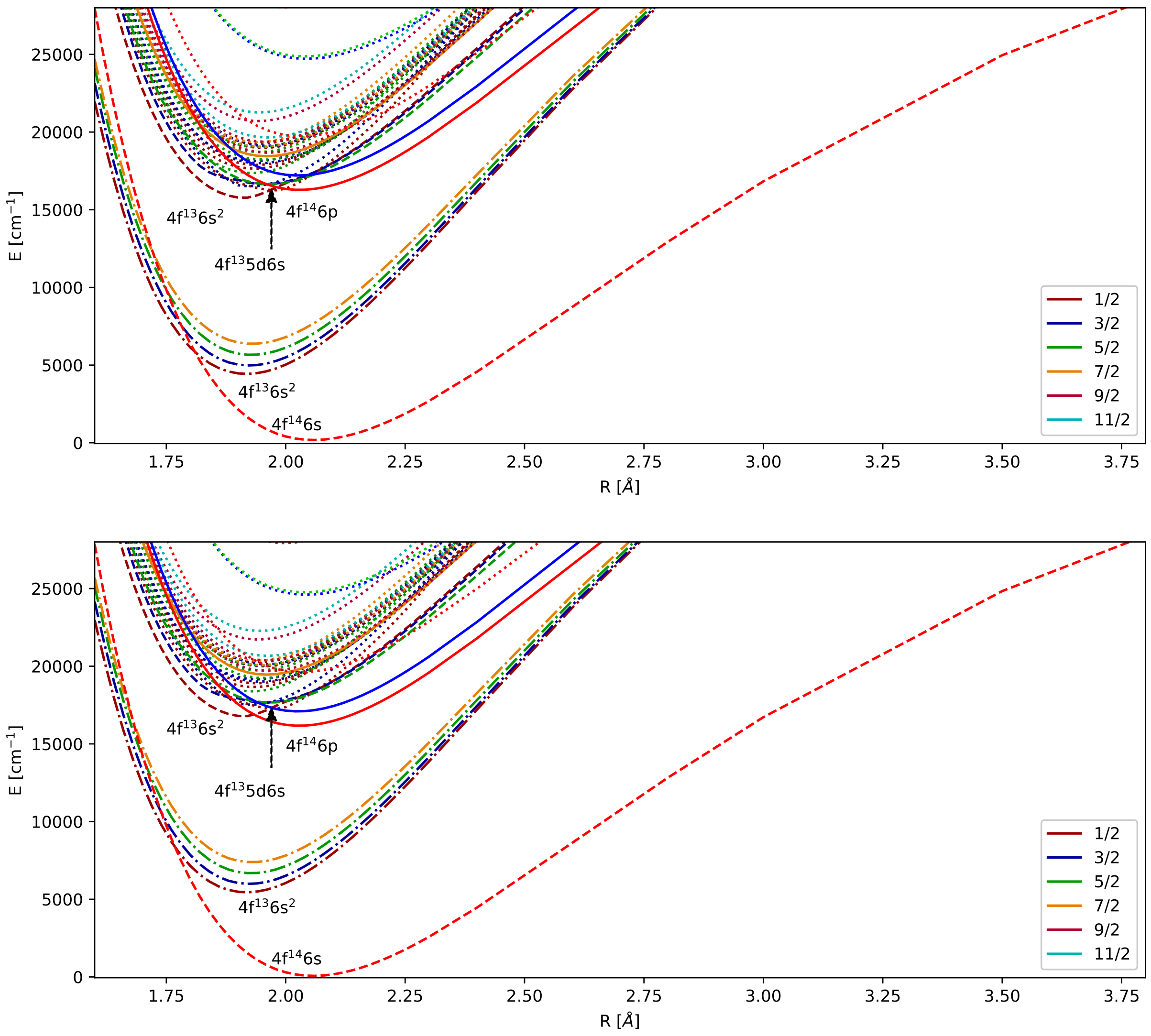

Figure S8: Combination of the sets of KRCI potentials. The lowest $\Omega = 1/2$ are denoted by the assigned dominant configuration. For the upper part the shift was applied at 15 Å, for the lower part at 10 Å.

The combination of the potential energy by shifting according to the outermost point is shown in figure S8. While the minimum of the potential changes from about 55000 to 51000 cm^{-1}, there are only small changes in the relative position of the minima of different electronic states.

Figure S9 shows the combined potentials energy curves for larger bond distances.

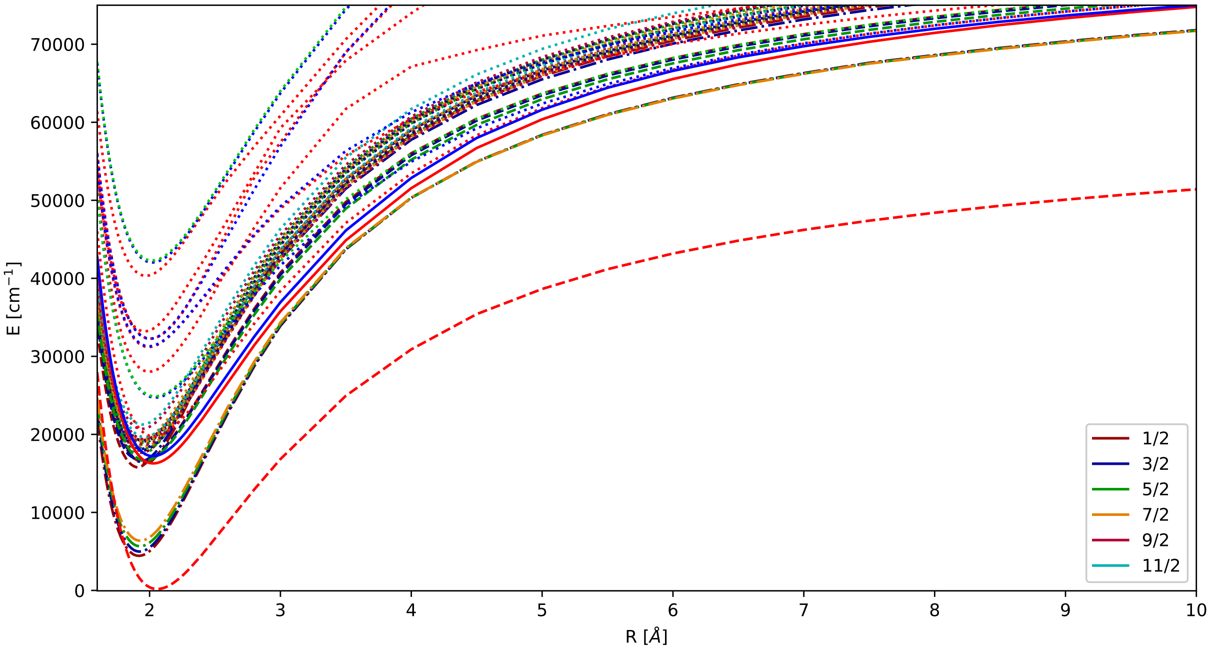

Figure S9: KRCI PECs for states with open and closed f-shell for extrapolation to the basis set limit.

2.7 Collection - transition dipole moments

Table S8: KRCI transition dipole moment table for the equilibrium distances of the lower state, corresponding to the TDMs displayed in figures S3 and S6. Values for the dyall.v4z basis set are listed.

Ω_L	state$_L$	Ω_U	state$_U$	|TDM| [e a_0]
$\frac{1}{2}$	1	$\frac{1}{2}$	3	0.403
$\frac{1}{2}$	1	$\frac{1}{2}$	6	2.900
$\frac{1}{2}$	1	$\frac{3}{2}$	2	3.684
$\frac{1}{2}$	1	$\frac{3}{2}$	5	0.111
$\frac{1}{2}$	2	$\frac{1}{2}$	4	0.010
$\frac{1}{2}$	2	$\frac{1}{2}$	5	0.034
$\frac{1}{2}$	2	$\frac{3}{2}$	1	0.036
$\frac{1}{2}$	2	$\frac{3}{2}$	3	0.076
$\frac{3}{2}$	1	$\frac{1}{2}$	4	0.010
$\frac{3}{2}$	1	$\frac{1}{2}$	5	0.067
$\frac{3}{2}$	1	$\frac{3}{2}$	3	0.071
$\frac{3}{2}$	1	$\frac{3}{2}$	4	0.004
$\frac{3}{2}$	1	$\frac{5}{2}$	1	0.058
$\frac{3}{2}$	1	$\frac{5}{2}$	2	0.037
$\frac{5}{2}$	1	$\frac{3}{2}$	3	0.037
$\frac{5}{2}$	1	$\frac{3}{2}$	4	0.001
$\frac{5}{2}$	1	$\frac{5}{2}$	2	0.070
$\frac{5}{2}$	1	$\frac{5}{2}$	3	0.021
$\frac{5}{2}$	1	$\frac{7}{2}$	1	0.066
$\frac{5}{2}$	1	$\frac{7}{2}$	2	0.035
$\frac{7}{2}$	1	$\frac{5}{2}$	2	0.001
$\frac{7}{2}$	1	$\frac{5}{2}$	3	0.034
$\frac{7}{2}$	1	$\frac{7}{2}$	1	0.059
$\frac{7}{2}$	1	$\frac{7}{2}$	2	0.026

3 Intermediate Hamiltonian Fock Space Coupled Cluster (IHFS-CCSD)

3.1 Closed f-shell - potential energy curves

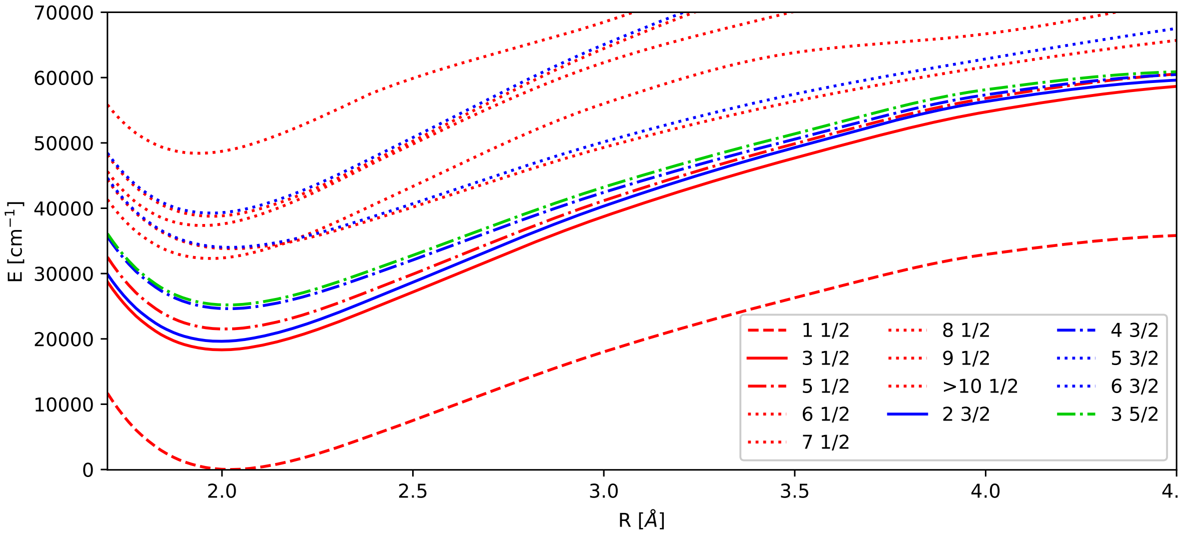

Figure S10: IHFS-CCSD PECs for states from the (0h,1p) sector with a cation reference for the quadruple zeta basis sets.

Table S9: Spectroscopic constants for the different electronic states ($\Omega = 1/2, 3/2, 5/2$) obtained by IHFS-CCSD for the (0h,1p) sector using double and triple zeta basis sets. Vibrational constant (ω_e), anharmonicty constant ($\omega_e\chi_e$), and transition energy (T_e), are given in cm^{-1}, the equilibrium bond distance (r_e) in Å.

Ω	state	v2z				v3z			
		r_e	ω_e	$\omega_e\chi_e$	T_e	r_e	ω_e	$\omega_e\chi_e$	T_e
1/2	1	2.0315	502	2.33	0	2.0281	504	2.32	0
1/2	2	2.0045	524	2.19	18610	2.0037	526	2.22	18425
1/2	3	2.0133	518	2.31	22113	2.0127	521	2.28	21687
1/2	4	2.0905	443	2.21	36083	1.9680	543	6.91	33550
1/2	5	1.9873	518	3.00	38956	2.0481	524	0.02	34724
1/2	6	1.9994	569	2.17	42974	1.9648	582	2.30	39207
1/2	7	2.0383	508	1.98	56216	1.9964	532	1.92	46115
1/2	8	1.8438	813	3.41	66635	1.9414	591	2.37	54196
3/2	1	2.0010	527	2.20	19980	2.0004	530	2.24	19747
3/2	2	2.0394	484	2.17	25904	2.0268	496	2.24	24977
3/2	3	2.0892	450	2.19	36389	2.0448	470	2.39	34962
3/2	4	2.0319	510	2.01	58253	1.9909	535	1.99	46891
5/2	1	2.0372	486	2.18	26612	2.0243	499	2.26	25563
ION		1.9506	594	2.08	48113	1.9483	597	2.15	48352

Table S10: Spectroscopic constants for the different electronic states ($\Omega = 1/2, 3/2, 5/2$) obtained by IHFS-CCSD for the (0h,1p) sector using double and triple zeta and extrapolation to the complete basis sets. Vibrational constant (ω_e), anharmonicty constant ($\omega_e\chi_e$), and transition energy (T_e), are given in cm^{-1}, the equilibrium bond distance (r_e) in Å.

Ω	state	v4z				CBS			
		r_e	ω_e	$\omega_e\chi_e$	T_e	r_e	ω_e	$\omega_e\chi_e$	T_e
1/2	1	2.0220	510	2.60	0	2.0176	515	2.82	0
1/2	2	1.9988	533	2.46	18323	1.9953	539	2.63	18249
1/2	3	2.0072	527	2.54	21506	2.0032	533	2.73	21375
1/2	4	1.9649	574	4.13	32306	1.9644	581	1.82	31416
1/2	5	2.0298	506	0.35	33748	2.0155	494	0.68	33018
1/2	6	1.9477	599	2.29	37343	1.9359	610	2.08	35954
1/2	7	1.9732	577	2.08	38770	1.9597	608	1.95	33366
1/2	8	1.9384	592	2.46	48402	1.9363	592	2.39	44179
3/2	1	1.9955	537	2.46	19629	1.9920	542	2.63	19543
3/2	2	2.0182	505	2.53	24624	2.0120	512	2.73	24363
3/2	3	2.0208	482	2.69	34007	2.0031	493	2.96	33271
3/2	4	1.9703	576	2.10	39254	1.9582	605	1.96	33646
5/2	1	2.0154	508	2.53	25193	2.0089	515	2.77	24919
ION		1.9450	604	1.98	48426	1.9437	609	1.86	49901

3.2 Open f-shell - potential energy curves

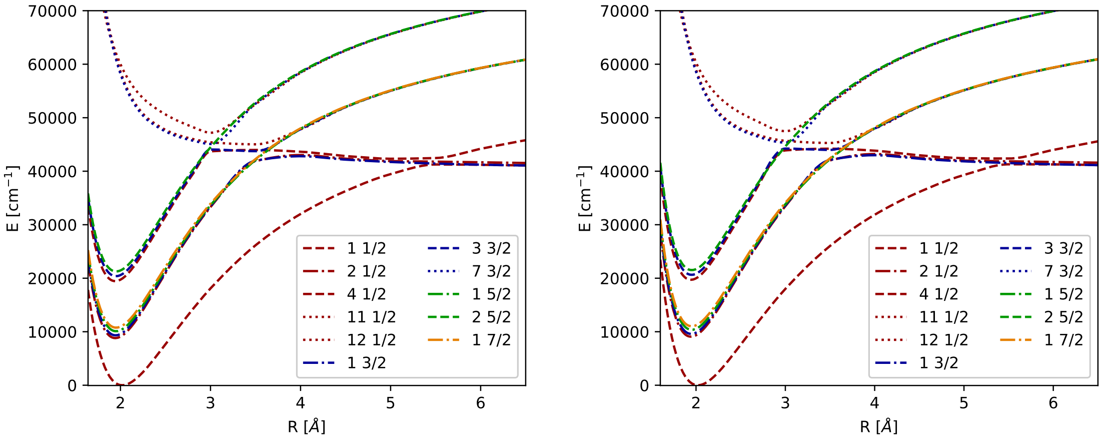

Figure S11: IHFS-CCSD PECs for (1h,0p) sector starting from the anion reference for the quadruple zeta basis sets and a shift of 0.2 and 0.1 Hartree, the extrapolation to 0.0 Hartree is shown in the figure below.

Table S11: Spectroscopic constants for the different electronic states ($\Omega = 1/2, 3/2, 5/2$) obtained by XIHFS-CCSD for the (1h,0p) sector using double and triple zeta basis sets. Vibrational constant (ω_e), anharmonicty constant ($\omega_e\chi_e$), and transition energy (T$_e$), are given in cm^{-1}, the equilibrium bond distance (r$_e$) in Å.

Ω	state	v2z				v3z			
		r$_e$	ω_e	$\omega_e\chi_e$	T$_e$	r$_e$	ω_e	$\omega_e\chi_e$	T$_e$
1/2	1	2.0239	497	2.06	0	2.0244	504	2.26	0
1/2	2	1.9481	619	3.17	4282	1.9463	598	2.81	7059
1/2	3	1.9410	605	2.73	14808	1.9444	594	2.72	17729
3/2	1	1.9440	602	2.73	4630	1.9479	591	2.73	7521
3/2	2	1.9478	599	2.75	15710	1.9518	588	2.72	18616
5/2	1	1.9488	597	2.74	5407	1.9530	586	2.72	8263
5/2	2	1.9491	592	2.73	16625	1.9533	581	2.71	19483
7/2	1	1.9487	591	2.73	6102	1.9529	581	2.71	8918
ION		2.1018	425	2.71	-8767	2.0935	430	2.66	-9418

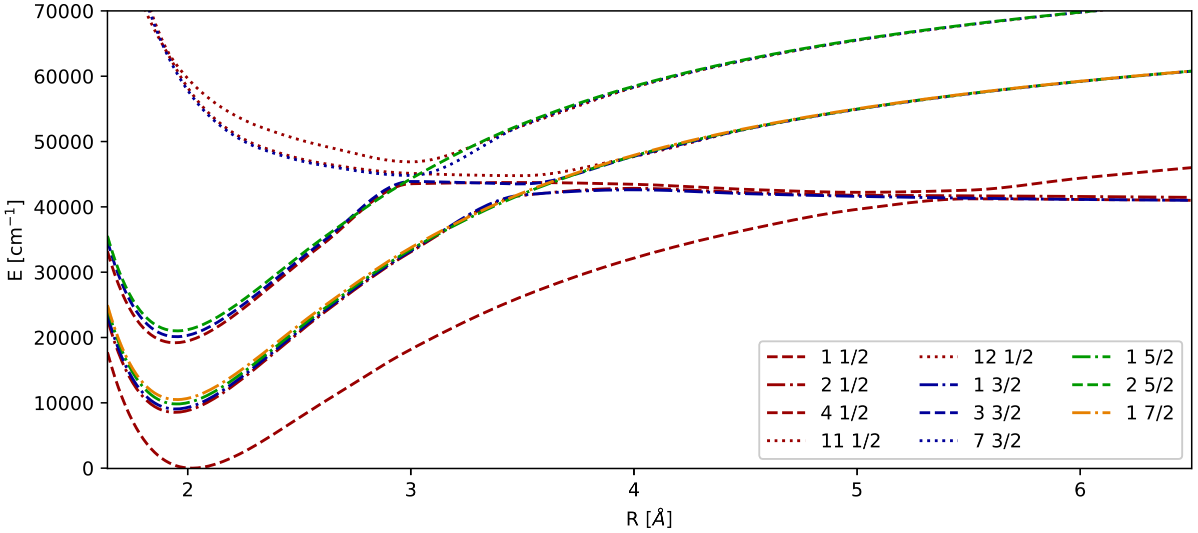

Figure S12: XIHFS-CCSD PECs for (1h,0p) sector starting from the anion reference for the quadruple zeta basis sets, extrapolation using the results in figure S11

Table S12: Spectroscopic constants for the different electronic states ($\Omega = 1/2, 3/2, 5/2$) obtained by XIHFS-CCSD for the (1h,0p) sector using quadruple zeta and extrapolation to the complete basis sets. Vibrational constant (ω_e), anharmonicty constant ($\omega_e\chi_e$), and transition energy (T_e), are given in cm^{-1}, the equilibrium bond distance (r_e) in Å.

Ω	state	v4z				CBS			
		r_e	ω_e	$\omega_e\chi_e$	T_e	r_e	ω_e	$\omega_e\chi_e$	T_e
1/2	1	2.0195	509	2.35	0	2.0159	513	2.42	0
1/2	2	1.9425	599	2.80	8543	1.9396	599	2.79	9627
1/2	3	1.9417	596	2.75	19195	1.9397	597	2.78	20267
3/2	1	1.9455	593	2.76	9057	1.9438	595	2.79	10180
3/2	2	1.9496	590	2.76	20121	1.9480	591	2.80	21222
5/2	1	1.9508	588	2.76	9825	1.9493	589	2.78	10968
5/2	2	1.9513	583	2.75	21011	1.9499	584	2.77	22127
7/2	1	1.9510	582	2.74	10494	1.9496	583	2.77	11645
ION		2.0851	435	2.74	-9579	2.0823	437	2.79	-8197

3.3 Combined potentials

Figure S13 shows the PECs for longer bond distances.

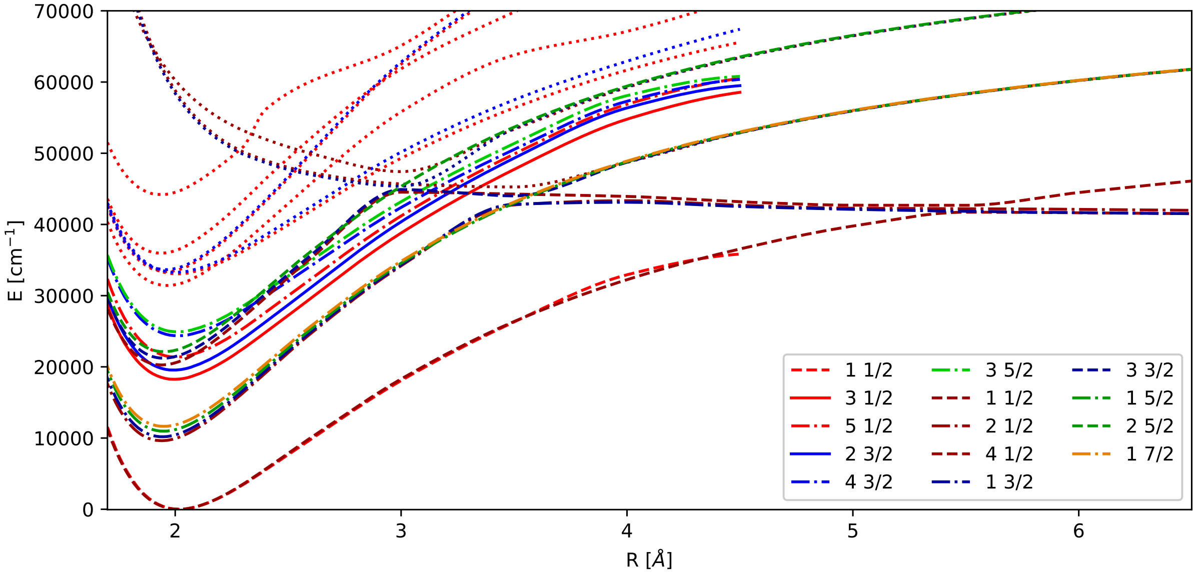

Figure S13: IHFS-CCSD PECs generated by the use of two different sectors and reference systems after extrapolation to the basis sets limit.

3.4 Spectroscopic quantities after mixing

We extracted the spectroscopic quantities for the adiabatized potentials shown in the main text and listed them here.

Table S13: Spectroscopic constants for the different electronic states ($\Omega = 1/2, 3/2, 5/2$) obtained by IHFS-CCSD after basis set extrapolation, results from two sectors are combined using adiabatization with different coupling constants (C). Vibrational constant (ω_e), anharmonicty constant ($\omega_e\chi_e$), and transition energy (T_e), are given in cm^{-1}, the equilibrium bond distance (r_e) in Å.

Ω	state	C = 10 cm^{-1}				C = 100 cm^{-1}				C = 1000 cm^{-1}			
		r_e	ω_e	$\omega_e\chi_e$	T_e	r_e	ω_e	$\omega_e\chi_e$	T_e	ω_e	$\omega_e\chi_e$	T_e	
1/2	1	2.0177	515	2.80	0	2.0177	515	2.80	0	2.0167	515	2.80	0
1/2	2	1.9397	599	2.77	9618	1.9397	599	2.77	9617	1.9418	596	2.76	9522
1/2	3	1.9953	539	2.61	18249	1.9952	538	2.60	18247	1.9843	533	2.35	18066
1/2	4	1.9337	609	8.95	20260	1.9349	603	8.61	20258	1.9654	561	3.65	20160
1/2	5	2.0035	586	0.25	21336	2.0019	586	0.38	21359	1.9860	577	2.10	22209
1/2	6	1.9644	581	1.83	31417	1.9644	581	1.83	31419	1.9645	580	1.96	31667
1/2	7	2.0149	470	0.14	33029	2.0149	470	0.14	33031	2.0132	472	0.24	33255
1/2	8	1.9647	633	2.62	33384	1.9647	633	2.62	33386	1.9656	628	2.61	33633
3/2	1	1.9438	594	2.76	10171	1.9438	594	2.76	10170	1.9447	593	2.76	10097
3/2	2	1.9921	542	2.61	19543	1.9919	542	2.60	19540	1.9820	541	2.50	19316
3/2	3	1.9480	591	2.77	21213	1.9482	591	2.80	21217	1.9621	580	3.13	21524
3/2	4	2.0121	512	2.72	24363	2.0121	512	2.69	24369	2.0059	528	2.33	24897
3/2	5	2.0023	473	1.95	33287	2.0023	473	1.95	33290	1.9998	476	2.25	33537
3/2	6	1.9608	632	3.13	33644	1.9608	632	3.13	33647	1.9630	623	2.67	33954
5/2	1	1.9493	589	2.75	10959	1.9493	589	2.75	10960	1.9496	588	2.76	11075
5/2	2	1.9500	584	2.75	22118	1.9500	583	2.76	22117	1.9542	572	3.01	22014
5/2	3	2.0090	515	2.75	24920	2.0089	516	2.72	24926	2.0024	532	2.38	25513

4 Equation-of-motion coupled cluster (EOM-CCSD)

4.1 Yb cation transition energies

Table S14: Transition energies for the Yb cation. Reference values have been obtained from the Nist database, the computed values were obtained for different basis sets with IP-EOM-CCSD or EA-EOM-CCSD coupled cluster.

		NIST[9]	EA			IP		
state	conf	E	2z	3z	4z	2z	3z	4z
$^2S_{1/2}$	$4f^{14}6s$	0	0	0	0	0	0	0
$^2F^\circ_{7/2}$	$4f^{13}6s^2$	21419				12054	13524	16092
$^2F^\circ_{5/2}$	$4f^{13}6s^2$	31568				22629	24139	26655
$^2D_{3/2}$	$4f^{14}5d$	22961	24209	24073	24060	32956	33306	33712
$^2D_{5/2}$	$4f^{14}5d$	24333	25457	25351	25341	33589	33865	34263
$^2P^\circ_{1/2}$	$4f^{14}6p$	27062	27780	27539	27857	31553	31938	32112
$^2P^\circ_{3/2}$	$4^{14}6p$	30392	31246	30954	31323	34621	34975	35147

4.2 Closed f-shell - potential energy curves

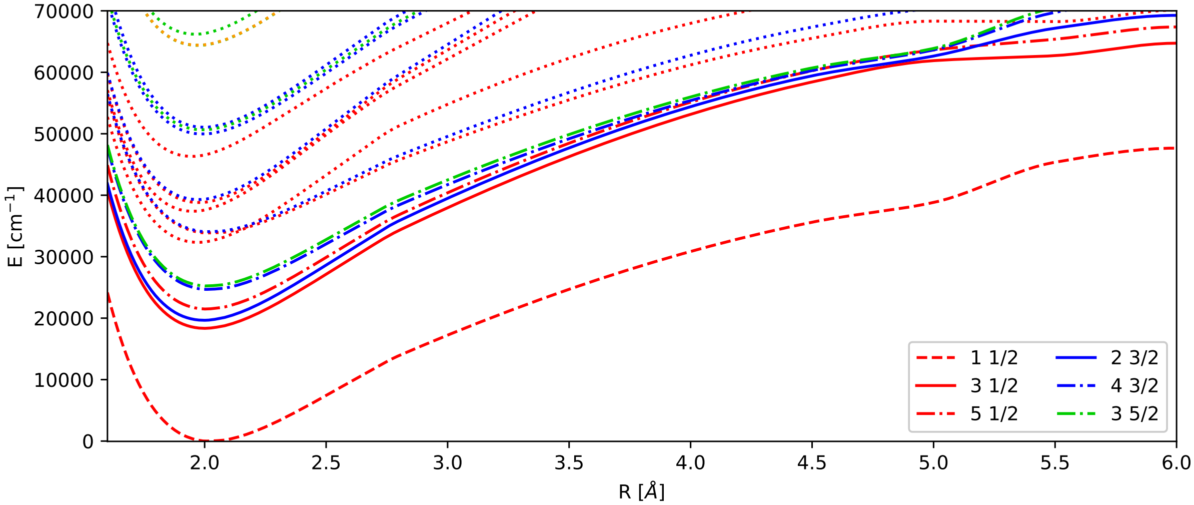

Figure S14: EOM-CCSD PECs for states from the (0h,1p) sector with a cation reference for the quadruple zeta basis sets.

Table S15: Spectroscopic constants for the different electronic states ($\Omega = 1/2, 3/2, 5/2$) obtained by EOM-CCSD for the (0h,1p) sector using different basis set sizes. Vibrational constant (ω_e), anharmonicty constant ($\omega_e\chi_e$), and transition energy (T_e), are given in cm^{-1}, the equilibrium bond distance (r_e) in Å.

Ω	state	v3z				v4z				CBS			
		r_e	ω_e	$\omega_e\chi_e$	T_e	r_e	ω_e	$\omega_e\chi_e$	T_e	r_e	ω_e	$\omega_e\chi_e$	T_e
1/2	1	2.0287	503	2.30	0	2.0248	508	2.57	0	2.0230	511	2.80	0
1/2	2	2.0040	525	2.22	18407	2.0012	532	2.52	18384	2.0004	536	2.72	18373
1/2	3	2.0128	521	2.27	21631	2.0093	527	2.58	21524	2.0079	532	2.78	21448
1/2	4	1.9718	513	8.14	33599	1.9646	567	4.96	32407	1.9726	593	0.60	31497
1/2	5	2.0408	552	0.10	34419	2.0334	510	0.28	33772	2.0294	479	0.29	33299
1/2	6	1.9652	580	2.32	39226	1.9499	596	2.33	37425	1.9405	607	2.20	36100
1/2	7	1.9899	550	1.95	43303	1.9753	575	2.10	38850	1.9667	593	2.19	35591
1/2	8	1.9482	577	2.53	51348	1.9460	582	2.56	46354	1.9456	584	2.58	42725
3/2	1	2.0006	529	2.23	19738	1.9979	535	2.52	19696	1.9971	540	2.72	19672
3/2	2	2.0273	496	2.25	24996	2.0210	503	2.56	24691	2.0177	509	2.78	24468
3/2	3	2.0481	470	2.30	34695	2.0237	480	2.71	34062	2.0068	489	3.03	33563
3/2	4	1.9862	549	1.97	44290	1.9724	575	2.13	39335	1.9646	593	2.14	35711
3/2	5	2.0073	525	2.10	60165	1.9900	550	2.33	50005	1.9797	569	2.45	42573
3/2	6	2.0096	539	2.15	62569	1.9961	563	2.39	51099	1.9881	581	2.54	42720
5/2	1	2.0248	498	2.25	25584	2.0181	507	2.56	25259	2.0146	513	2.80	25023
5/2	2	2.0085	523	2.10	61177	1.9905	548	2.34	50686	1.9796	567	2.48	43011
5/2	3	1.9834	549	2.24	75514	1.9711	569	2.39	64418	1.9642	581	2.34	56321
5/2	4	1.9526	583	2.56	77193	1.9601	582	2.24	66234	1.9659	583	2.18	58242
7/2	1	1.9834	549	2.24	75566	1.9712	569	2.39	64446	1.9643	581	2.34	56331
7/2	2	1.9499	582	2.45	77694	1.9463	590	2.48	80882	1.9006	708	45.10	83343
ION	0	1.9485	596	2.16	48387	1.9471	604	2.02	48471	1.9471	609	1.91	48578

4.3 Open f-shell - potential energy curves

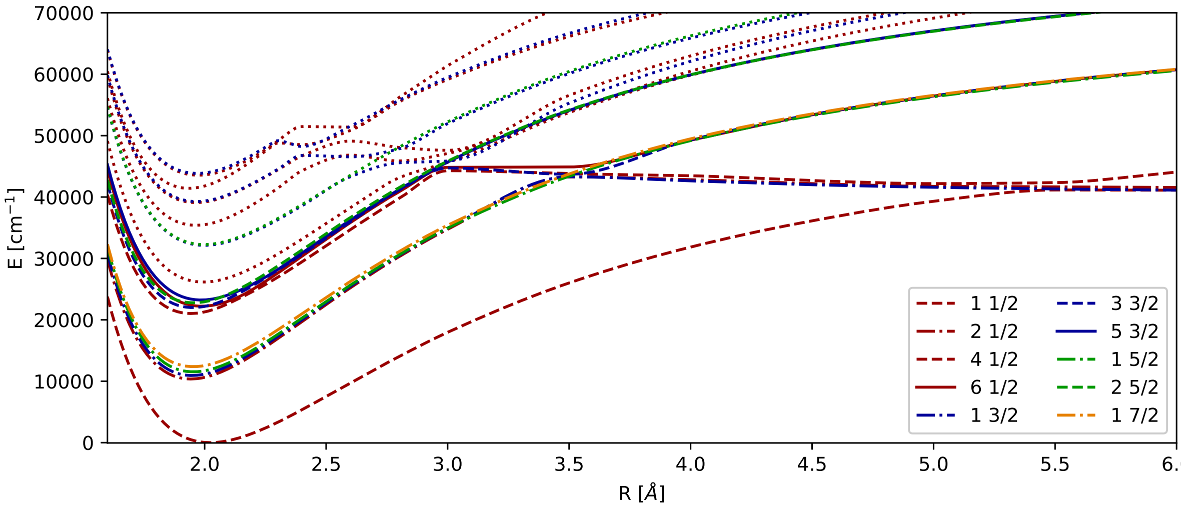

Figure S15: EOM-CCSD PECs for states from the (1h, 0p) sector with a anion reference for the quadruple zeta basis sets.

Table S16: Spectroscopic constants for the different electronic states ($\Omega = 1/2, 3/2, 5/2$) obtained by EOM-CCSD for the (1h,0p) sector using different basis set sizes. Vibrational constant (ω_e), anharmonicty constant ($\omega_e\chi_e$), and transition energy (T_e), are given in cm^{-1}, the equilibrium bond distance (r_e) in Å.

Ω	state	v3z				v4z				CBS			
		r_e	ω_e	$\omega_e\chi_e$	T_e	r_e	ω_e	$\omega_e\chi_e$	T_e	r_e	ω_e	$\omega_e\chi_e$	T_e
1/2	1	2.0247	503	2.27	0	2.0233	507	2.41	0	2.0250	508	2.53	0
1/2	2	1.9462	597	2.78	7380	1.9431	595	2.67	10361	1.9432	591	2.59	12568
1/2	3	1.9445	593	2.70	18046	1.9428	588	3.23	21029	1.9432	582	4.06	23241
1/2	4	1.9922	548	2.31	22306	1.9889	564	1.51	22205	1.9886	573	1.18	22147
1/2	5	1.9991	543	2.36	26384	1.9941	549	2.39	26151	1.9931	551	2.40	25991
1/2	6	1.9735	571	2.21	36936	1.9645	584	2.21	35391	1.9606	591	2.18	34280
1/2	7	1.9973	524	2.77	40983	1.9634	567	2.82	39091	1.9441	595	2.76	37639
1/2	8	1.9506	609	2.41	43943	1.9346	620	2.33	41412	1.9252	625	2.24	39575
3/2	1	1.9482	590	2.71	7848	1.9475	590	2.66	10931	1.9494	588	2.61	13211
3/2	2	1.9521	587	2.72	18941	1.9516	586	2.68	21994	1.9537	584	2.64	24251
3/2	3	1.9889	551	2.31	23370	1.9855	556	2.30	23227	1.9857	557	2.28	23137
3/2	4	2.0100	522	2.37	32680	1.9987	533	2.47	32132	1.9936	539	2.45	31734
3/2	5	2.0017	523	2.70	41181	1.9659	564	2.79	39262	1.9451	591	2.75	37778
5/2	1	1.9532	585	2.72	8588	1.9530	584	2.66	11529	1.9553	582	2.61	13703
5/2	2	1.9536	580	2.71	19807	1.9535	579	2.65	22768	1.9559	577	2.62	24957
5/2	3	2.0085	524	2.39	32839	1.9974	534	2.46	32257	1.9923	540	2.45	31834
7/2	1	1.9532	580	2.71	9245	1.9532	579	2.64	12373	1.9556	577	2.62	14685
ION	0	2.0941	429	2.64	-9522	2.0889	431	2.99	- 9713	2.0883	432	3.25	-9876

4.4 Combined potentials

Figure S16 shows the PECs for longer bond distances.

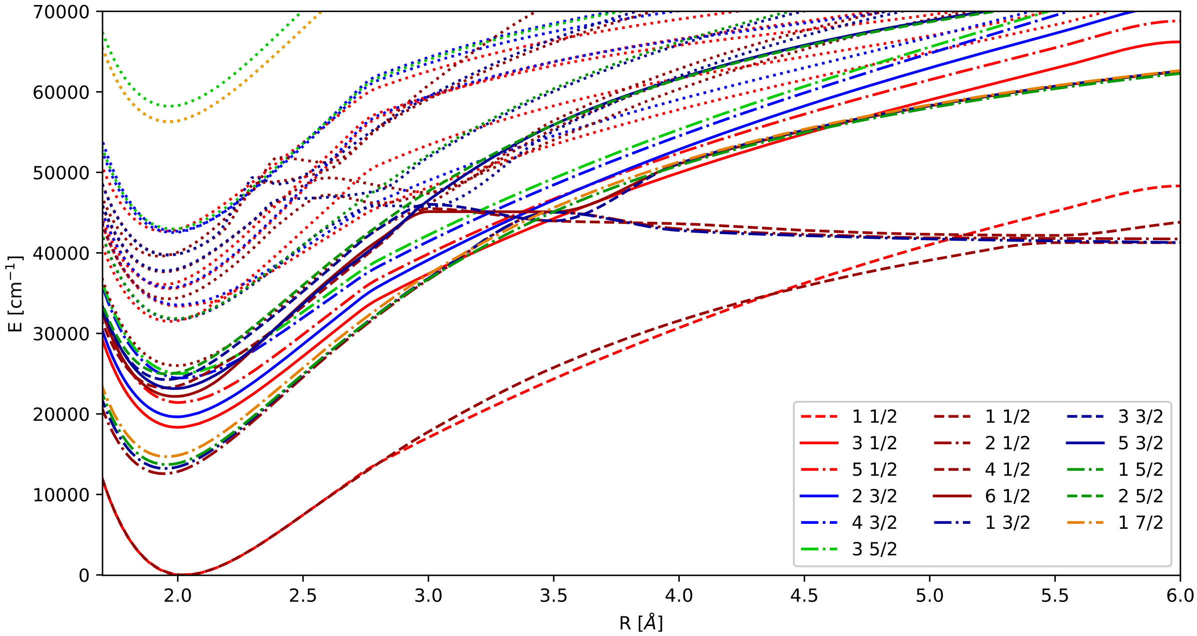

Figure S16: EOM-CCSD PECs generated by the use of two different sectors and reference systems for extrapolation to the basis set limit.

5 Spectroscopic parameters

Table S17 contains the complete list of calculated states, of which a selection was presented in table 7 in the main manuscript.

Table S17: Spectroscopic constants for excited states with $\Omega = 1/2, 3/2, 5/2$, starting from 18000 cm^{-1} for different methods using the values after extrapolation to the basis set limit. Transition energy (T_e), vibrational constant (ω_e), and anharmonicty constant ($\omega_e\chi_e$) are given in cm^{-1}, the equilibrium bond distance (r_e) in Å. Experimental transitions that were not assigned(n.a.) are also listed.

Ω	method	state	configuration	T_e	r_e	ω_e	$\omega_e\chi_e$
1/2	KRCI[a]	3	$4f^{13}\sigma_{6s}^2$	15572	1.9038	655	13.57
		4	$4f^{13}5d\sigma_{6s}^1$	16020	1.9603	620	0.08
		5	$4f^{14}6p^1$	16189	2.0504	496	2.38
		6	$4f^{13}5d^1\sigma_{6s}^1$	17350	1.9360	598	2.50
		7	$4f^{13}5d^1\sigma_{6s}^1$	17587	1.9417	592	2.56
		8	$4f^{13}5d^1\sigma_{6s}^1$	18675	1.9539	582	2.71
		9	$4f^{13}5d^1\sigma_{6s}^1$	18952	1.9479	578	2.71
		10	$4f^{14}5d^1$	19631	2.0552	490	2.49
	EOM-CCSD	3	$4f^{14}6p^1$	18373	2.0004	536	2.72
		4	$4f^{14}5d^1$	21448	2.0079	532	2.78
		5	$4f^{14}6p^1$	22147	1.9886	573	1.18
		6	$4f^{13}\sigma_{6s}^2$	23241	1.9432	582	4.06
	IHFS-CCSD	3	$4f^{14}6p^1$	18249	1.9953	539	2.63
		4	$4f^{13}\sigma_{6s}^2$	20267	1.9397	597	2.78
		5	$4f^{14}5d^1$	21375	2.0032	533	2.73
	MRCI[10]		$4f^{13}\sigma_{6s}^2$		1.948	600	
	exp.[11] [a]	3		18106.20		537	3
	exp.[11]	4	$[18.6]_{1/2}$	18705.06			

Continued on next page

Table S17 – *Continued from previous page*

Ω	method	state	configuration	T$_e$	r$_e$	ω_e	$\omega_e\chi_e$
	exp.[12]		[557]	18574	1.9656	502.15	
	exp.[12]		[561]	18699	1.9571		
3/2	KRCI[a]	2	$4f^{13}\sigma_{6s}^2$	16206	1.9331	711	8.45
		3	$4f^{13}5d^1\sigma_{6s}^1$	16346	1.9505	516	0.80
		4	$4f^{14}6p^1$	17123	2.0473	499	2.37
		5	$4f^{13}5d^1\sigma_{6s}^1$	17693	1.9473	585	2.47
		6	$4f^{13}5d^1\sigma_{6s}^1$	17843	1.9408	584	3.30
		¿7	$4f^{14}5d^1$	24583	2.0669	470	2.50
	EOM-CCSD	2	$4f^{14}6p^1$	19672	1.9971	540	2.72
		3	$4f^{14}6p^1$	23137	1.9857	557	2.28
		4	$4f^{13}\sigma_{6s}^2$	24251	1.9537	584	2.64
		5	$4f^{14}5d^1$	24468	2.0177	509	2.78
	IHFS-CCSD	2	$4f^{14}6p^1$	19543	1.9920	542	2.63
		3	$4f^{13}\sigma_{6s}^2$	21222	1.9480	591	2.80
		4	$4f^{14}5d^1$	24363	2.0120	512	2.73
	MRCI[10]		$4f^{13}\sigma_{6s}^2$		1.953	596	
	exp.[11]	2		19471.49			
5/2	KRCI[a]	2	$4f^{13}5d^1\sigma_{6s}^1$	16340	1.9677	564	2.42
		3	$4f^{13}\sigma_{6s}^2$	17063	1.9302	635	1.41
		4	$4f^{13}5d^1\sigma_{6s}^1$	17700	1.9627	571	0.93
		5	$4f^{13}5d^1\sigma_{6s}^1$	18422	1.9451	584	3.07
		¿6	$4f^{14}5d^1$	24744	2.0639	474	2.48

Continued on next page

Table S17 – *Continued from previous page*

Ω	method	state	configuration	T_e	r_e	ω_e	$\omega_e\chi_e$
	EOM-CCSD	2	$4f^{13}\sigma_{6s}^2$	24957	1.95359	577	2.62
		3	$4f^{14}5d^1$	25023	2.0146	513	2.80
	IHFS-CCSD	2	$4f^{13}\sigma_{6s}^2$	22127	1.9499	584	2.77
		3	$4f^{14}5d^1$	24919	2.0089	515	2.77
	MRCI [10]		$4f^{13}\sigma_{6s}^2$		1.954	590	
n.a.	exp. [13]		[574]	19150			
	exp. [13]		[578]	19280			
	exp. [14]		C_1	23035.3		523	2
	exp. [14]		C_2	23256.0		507	2
	exp. [14]		D	26014.8		574.6	2.8

[a] KRCI transition energies for the $4f^{13}$ sector were obtained by adding 4144 cm^{-1}, an estimate for the energy of the lowest state in this manifold.

6 Frank-Condon factors (FCFs) for different methods

Frank-Condon factors are shown in figure S17 for MRCI, EOM-CCSD and IHFS-CCSD. From it, we observe a marked difference between the configuration interaction and coupled cluster results: first, the equilibrium distance and curvature are quite different, something that, in turn, change the overlap and resulting Frank-Condon factors as well as the progression of the vibrational levels.

Additionally, there is more uncertainty with regards to the position of the potential energy curves, as configuration interaction is not size consistent and we observe transition in the range from 14000 to 19000 cm^{-1} for these states. The states are more dense is this case, which is expected as states of the $Yb(4f^{13}[F^{\circ}_{7/2}]5d^1\sigma^1_{6s})F$ configuration are included, which

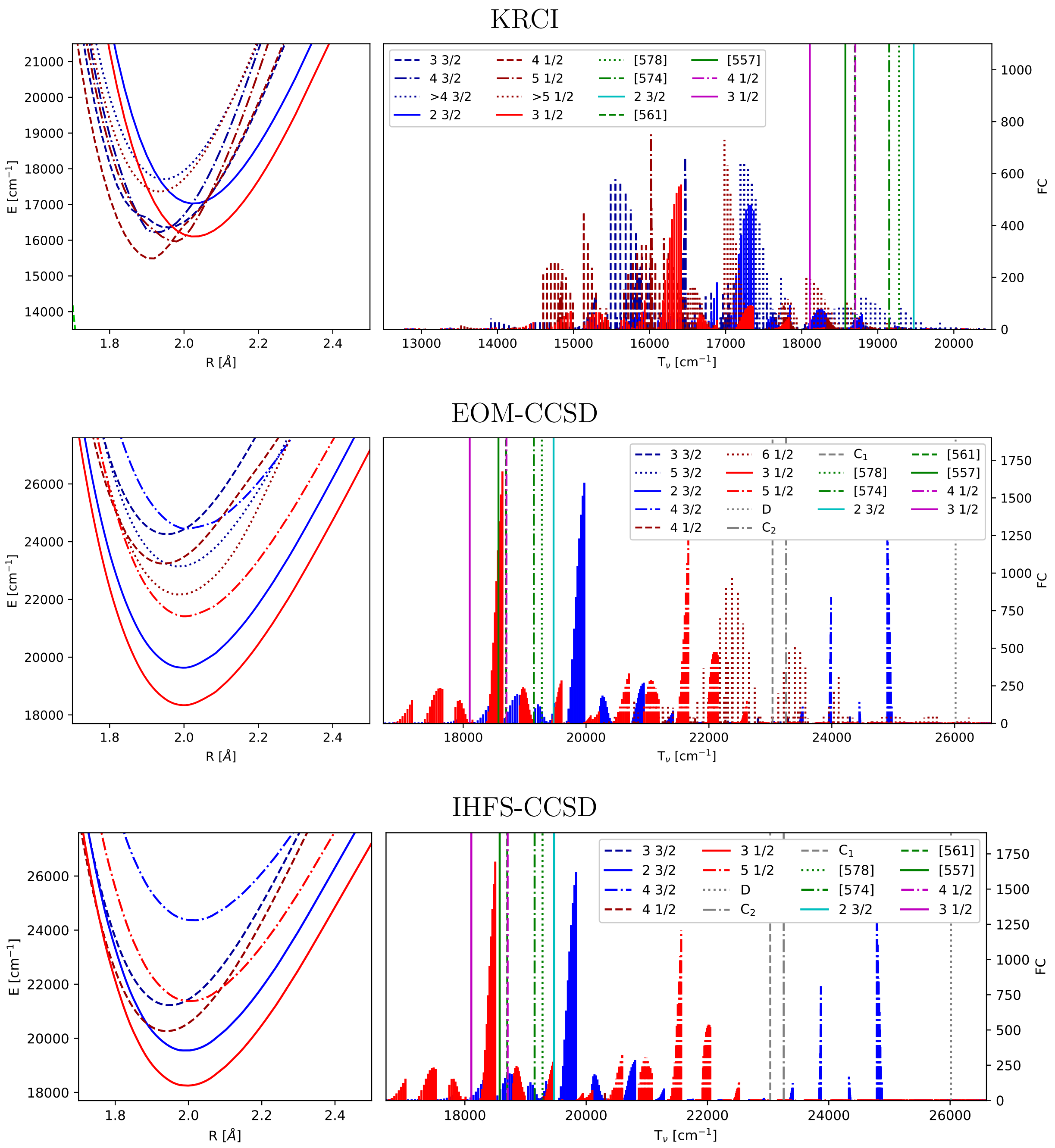

Figure S17: Frank-Condon factors obtained with the LEVEL program[15] by using the potential energy curves on the left, which have been obtained by different ab-intio methods. The lowest 10 vibrational levels of the ground state were used as well as the lowest 60 vibrational levels of the excited state. The experimental values have been added as straight lines.[11–13]

are missing in the coupled cluster descriptions.

The EOM-CCSD and IHFS-CCSD Frank-Condon factors are quite similar to each other, with the exception of the additional $6_{1/2}$ state in EOM-CCSD, probably stemming from the contributions by (2h,1p)/(1h,2p) to the final states. The results for the $3_{1/2}$ and $2_{3/2}$ state are close to the experimentally observed positions, only about 500 cm^{-1} too high, and show the same splitting. The transition of the $5_{1/2}$ states are also similar in both coupled cluster treatments.

References

(1) Pototschnig, J. V.; Dyall, K. G.; Visscher, L.; André Severo Pereira Gomes, Electronic Spectra of Ytterbium Fluoride from Relativistic Electronic Structure Calculations. 2021; https://arxiv.org/abs/2107.11234v1.

(2) Thyssen, J. Development and Applications of Methods for Correlated Relativistic Calculations of Molecular Properties. Ph.D. thesis, University of Southern Denmark, 2001.

(3) Cox, P. A. Ionization energies of open-shell molecules in the frozen orbital approximation. *null* **1975**, *30*, 389–403.

(4) Sikkema, J.; Visscher, L.; Saue, T.; Iliaš, M. The molecular mean-field approach for correlated relativistic calculations. *J. Chem. Phys.* **2009**, *131*, 124116.

(5) Ilias, M.; Saue, T. An infinite-order two-component relativistic Hamiltonian by a simple one-step transformation. *J. Chem. Phys.* **2007**, *126*, 064102.

(6) Schimmelpfennig, B. *AMFI, an atomic mean-field spin-orbit integral program*. University of Stockholm, Stockholm, Sweden; 1999.

(7) Visscher, L. Approximate molecular relativistic Dirac-Coulomb calculations using a simple Coulombic correction. *Theoretical Chemistry Accounts* **1997**, *98*, 68–70.

(8) Kramida, A.; Yu. Ralchenko,; Reader, J.; and NIST ASD Team, NIST Atomic Spectra Database (ver. 5.8), [Online]. Available: https://physics.nist.gov/asd [2017, April 9]. National Institute of Standards and Technology, Gaithersburg, MD., 2020.

(9) Huber, K.; Herzberg, G. Constants of Diatomic Molecules. National Institute of Standards and Technology, Gaithersburg MD, 20899, 2005; (data prepared by J.W. Gallagher and R.D. Johnson, III) in NIST Chemistry WebBook, NIST Standard Reference Database Number 69, Eds. P.J. Linstrom and W.G. Mallard.

(10) Dolg, M.; Stoll, H.; Preuss, H. Abinitio Pseudopotential Study of Ybh and Ybf. *Chemical Physics* **1992**, *165*, 21–30.

(11) Dunfield, K. L.; Linton, C.; Clarke, T. E.; McBride, J.; Adam, A. G.; Peers, J. R. D. Laser spectroscopy of the lanthanide monofluorides: Analysis of the A(2)Pi-X(2)Sigma(+) transition of ytterbium monofluoride. *Journal of Molecular Spectroscopy* **1995**, *174*, 433–445.

(12) Lim, J.; Almond, J. R.; Tarbutt, M. R.; Nguyen, D. T.; Steimle, T. C. The [557]-X-2 Sigma(+) and [561]-X-2 Sigma(+) bands of ytterbium fluoride, (YbF)-Yb-174. *Journal of Molecular Spectroscopy* **2017**, *338*, 81–90.

(13) Smallman, I. J.; Wang, F.; Steimle, T. C.; Tarbutt, M. R.; Hinds, E. A. Radiative branching ratios for excited states of 174YbF: Application to laser cooling. *Spectroscopic Tests of Fundamental Physics* **2014**, *300*, 3–6.

(14) Uttam, K. N.; Joshi, M. M. A New Band System of the YbF Molecule. *Journal of Molecular Spectroscopy* **1995**, *174*, 290–296.

(15) Le Roy, R. J. LEVEL: A computer program for solving the radial Schrödinger equation for bound and quasibound levels. *Journal of Quantitative Spectroscopy and Radiative Transfer* **2017**, *186*, 167–178.
